# A synthetic angiotensin II/ACE2-based hormone shunt controlling experimental hypertension

**DOI:** 10.1038/s41467-026-71796-z

**Published:** 2026-04-11

**Authors:** Gokberk Unal, Maysam Mansouri, Yu-Qing Xie, Christian Mueller, Ghislaine Charpin-El Hamri, Martin Fussenegger

**Affiliations:** 1 Department of Biosystems Science and Engineering, ETH Zurich, Basel, Switzerland; 2 Department of Cardiology, University Hospital Basel, Basel, Switzerland; 3 Département Génie Biologique, Institut Universitaire de Technologie, Lyon, France; 4 Faculty of Science, University of Basel, Basel, Switzerland

**Keywords:** Synthetic biology, Synthetic biology, Hypertension

## Abstract

Arterial hypertension is the leading preventable cause of various life-threatening medical conditions and comorbidities that affect over 1.3 billion adults, causing over 7.5 million annual deaths worldwide. Capitalizing on a synthetic biology-inspired engineering approach, we design a fully human, antihypertensive gene circuit called ARCH (autonomous regulator of chronic hypertension), which precisely monitors and efficiently controls angiotensin-dependent, renin-angiotensin system (RAS)-driven hypertension, in which angiotensin II (Ang-II) is persistently elevated, in a reversible, self-sufficient, and closed-loop manner. We utilize the ectopically expressed native type-1 angiotensin receptor (AT_1_R) to monitor the blood level of the hypertension biomarker Ang-II. We then rewire the AT_1_R activation during hypertension to a synthetic promoter to coordinate the expression of clinically licensed, secretion-engineered, soluble therapeutic angiotensin-converting enzyme 2 (stACE2), which degrades Ang-II and restores normotension. Implantation of microencapsulated ARCH-transgenic human cells into male hypertensive mice restores and maintains normal blood pressure.

## Abstract (web summary)

Chronic diseases require lifelong maintenance. Here, the authors present ARCH, a closed-loop genetic circuit that autonomously maintains Angiotensin-II homeostasis, demonstrating translational potential against RAS-dependent hypertension.

## Introduction

Arterial hypertension is a multifactorial medical condition involving dysfunction of the renin-angiotensin system (RAS), which fine-tunes blood pressure, fluid and electrolyte balance, and systemic vascular resistance^1,2^. Lifelong treatment is required to prevent a range of life-threatening complications^3^ such as acute aortic syndromes^4^, acute myocardial infarction^5^, and stroke^5^, as well as a variety of comorbidities including metabolic syndrome^6^, blindness^7^, diabetes^8^, and dementia^9^. Arterial hypertension currently affects over 30% of the global population of individuals above the age of 50 years, having dramatically increased since the mid-2000s^10^, and is the major cause of death and disability throughout the world^2^. Since persistent high blood pressure develops slowly over time, often without obvious symptoms, arterial hypertension remains undiagnosed in almost 40% of the population, developing into a “silent killer”^11^. Current treatment options are limited to lifelong daily use of antihypertensive prescription drugs^12^ including calcium channel blockers (e.g., nifedipine), angiotensin II receptor blockers (e.g., valsartan), diuretics (e.g., chlorthalidone), angiotensin-converting enzyme (ACE) inhibitors (e.g., enalapril), and beta blockers (e.g., atenolol). Unfortunately, each class of drugs has severe contraindications, dosing challenges, and multiple side effects, including asthma, miscarriage, and diabetes^13^, and a single drug is often not sufficient to restore normal blood pressure. Thus, despite the availability of a wide range of licensed antihypertensive drugs that generate over 25 billion USD in revenue annually^14^, over 80% of patients do not have their blood pressure under good control^2^ and medication adherence remains below 50%^15^.

Consequently, new antihypertensive treatment strategies are urgently needed. This is particularly challenging, since blood-pressure homeostasis is a complex process intricately fine-tuned by the RAS^1^. When blood flow is reduced, or β1-adrenergic receptors are stimulated, the proprotein convertase furin in juxtaglomerular cells of the kidney activates endogenous systemic prorenin, releasing renin into the circulation^16^. In the bloodstream, renin converts liver-released angiotensinogen into the decapeptide angiotensin I, which is cleaved by ACE on the surface of vascular endothelial cells to generate the octapeptide angiotensin II (Ang-II)^17^. Ang-II activates type-1 angiotensin receptor (AT_1_R), which mediates a potent vasoconstrictive effect, resulting in an increase in blood pressure^18^. Ang-II also triggers the delivery of aldosterone, which increases the blood pressure by promoting the reabsorption of sodium and release of potassium^19^.

Blood pressure homeostasis is maintained by the action of angiotensin-converting enzyme 2 (ACE2)^18^. ACE2 is present in membrane-bound form (mACE2) on many cells in the intestines, kidney, and heart until it is converted to soluble ACE2 (sACE2) by the enzyme ADAM17^20^. sACE2 indirectly lowers the blood pressure by catalyzing the hydrolysis of Ang-II to angiotensin 1–7, a vasodilator that directly decreases blood pressure by binding to MasR receptors^21^. mACE2 and sACE2 have recently attracted attention as drug targets and decoy therapeutics for emergency Covid-19 treatment since mACE2 interacts with the spike protein of many coronaviruses, including SARS-CoV-2, and serves as their cellular entry point^22,23^.

Despite the development of many medications that interfere with the vasoconstrictive or vasorelaxant axes of the RAS to maintain blood pressure within the optimal range^24^, dosage and timing must be carefully controlled, and poor patient compliance can have severe consequences^25^. Indeed, dosing and medication adherence remain major concerns in the long-term management of many chronic disorders^15,26^. One approach to overcome these issues is to develop cell-based therapies using implanted cells that dynamically rewire biomarker sensing to secretion of protein therapeutics in a closed-loop manner. Indeed, such devices have the potential to be foundation stones of future personalized medicine^27–30^. Specifically, genetic closed-loop systems in which critical blood biomarker concentrations dynamically manage the release of biopharmaceuticals that attenuate the medical condition and decrease the biomarker load would be able to autonomously restore and maintain a healthy homeostatic state. Closed-loop systems have already been successfully validated for the treatment of experimental arthritis^31,32^, obesity^33^, and diabetes^34–36^.

While Ang-II is not a universal biomarker of hypertension and Ang-II-independent forms exist^37,38^, ARCH is designed to target angiotensin-dependent hypertension, in which pathological Ang-II elevation can persist over extended periods across diverse etiologies (e.g., genetic predisposition, lifestyle, obesity, trauma)^39^. Moreover, the well-known inter-individual variability in Ang-II dynamics motivated a closed-loop precision-therapy design, in which the feedback controller autonomously self-tunes therapeutic output to patient-specific needs. Likewise, owing to its central role in vasorelaxant counter-regulation via enzymatic cleavage of Ang-II, soluble ACE2 (sACE2) is an attractive antihypertensive protein therapeutic whose clinical safety and dosing have been evaluated in humans, including studies in patients with COVID-19^40^. Notably, elevated Ang-II has also been implicated in pathophysiological processes beyond hypertension, including cardiovascular remodeling^41^, atherogenesis^42^, renal fibrosis^43^, insulin resistance^44^, and tumor angiogenesis^45^, underscoring the broader relevance of Ang-II homeostasis. Here, we describe a fully human, antihypertensive gene circuit called ARCH (autonomous regulator of chronic hypertension), which automatically restores and maintains normal blood pressure levels in mice suffering from angiotensin-dependent hypertension through modulation of Ang-II homeostasis by dynamically rewiring ectopic AT_1_R-mediated Ang-II sensing to a chimeric promoter driving the expression of secretion-engineered, stabilized ACE2 (stACE2).

## Results

### Design and validation of a human antihypertensive sensor-effector shunt

**Fig. 1:**
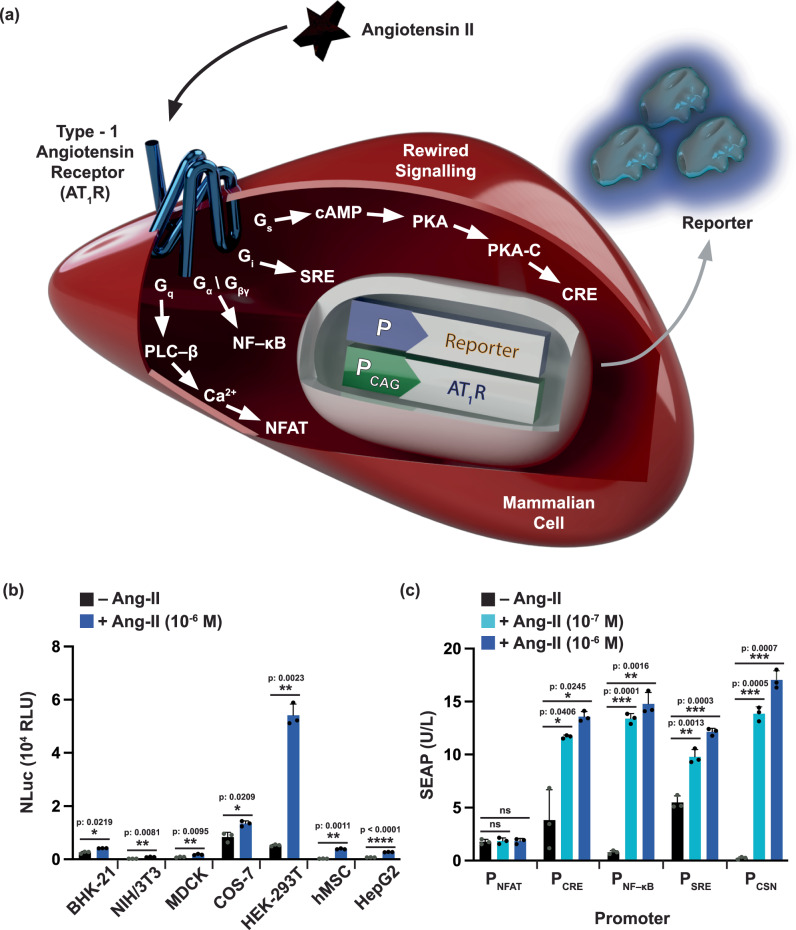
Establishing an Ang-II-dependent hypertension sensor in mammalian cells. **a** The 8-amino-acid peptide hormone Ang-II endogenously interacts with the receptor AT_1_R within the renin angiotensin system (RAS), leading to elevation of blood pressure. In our designer cell circuit, we rewire this interaction to trigger the expression of a reporter gene. **b** The sensor circuit was implemented in a variety of rodent (BHK-21, NIH/3T3), canine (MDCK), primate (COS-7), and human (HEK-293T, hMSC, HepG2) cells. Signaling was functional in all cell lines examined, but optimal transcriptional control was achieved with HEK-293T cells. **c** Various synthetic promoters were tested with AT_1_R (pGU52). The engineered promoter CSN containing triple sequence repeats of SRE, NFAT, and CRE (pGU23, P_CSN_-SS-nLuc-Fc-pA) produced the highest expression level as well as the tightest control. In (**b**, **c**): data shown as mean ± SD, *n* = 3 biological replicates, statistical analyses represent unpaired, two-tailed Welch’s *t*-test with a 95% confidence interval, where ns not significant, *p < 0.05, **p < 0.01, ***p < 0.001, ****p < 0.0001. Source data for this figure is available in the Source data file.

The binding of Ang-II to the G-protein-coupled receptor (GPCR) AT_1_R serves as the central control unit in the vasoconstrictive axis of the RAS^18^. Therefore, Ang-II is a key biomarker, and AT_1_R is a native biosensor of arterial hypertension^39^. Upon binding of Ang-II, AT_1_R interacts with endogenous G-proteins (G_sɑ_, G_qɑ/11_, G_ɑ12/13_) to activate the cyclic adenosine monophosphate (cAMP), Ca^2+^, and Rho GTPase-dependent intracellular signaling cascades^46^. These in turn can be rerouted to a synthetic promoter (P_CSN_, CRE-SRE-NFAT-P_min_) driving therapeutic interventions such as the expression of a desired protein therapeutic (Fig. 1a). To endow human cells with Ang-II sensor capacity, we ectopically expressed human AT_1_R in human embryonic kidney cells (HEK-293T) using a constitutive mammalian expression vector (pGU52, P_CAG_-hAT_1_R-pA). hAT_1_R is exclusively localized in the plasma membrane of transfected HEK-293T cells, and binding of Ang-II triggers the cAMP signaling pathway and activates synthetic promoters such as P_CSN_ (Supplementary Fig. 1). Indeed, upon co-transfecting the constitutive AT_1_R expression vector (pGU52, P_CAG_-hAT_1_R-pA) and a reporter expression vector encoding P_CSN_-driven stabilized secretion-engineered luciferase (pGU23, P_CSN_-SS-nLuc-Fc-pA), Ang-II-inducible reporter gene expression was observed in a variety of rodent (BHK-21, NIH/3T3), canine (MDCK), primate (COS-7), and human (HEK-293T, hMSC, HepG2) cells, indicating that AT_1_R-mediated Ang-II sensing is functional in a wide variety of mammalian cell types and species, which is crucial for translational potential (Fig. 1b). Since the Ang-II sensor performed most robustly in HEK-293T cells, we used this human cell line for all subsequent experiments to establish an all-human ARCH circuit. The comparatively strong response in HEK-293T cells likely reflects cell-type-specific factors, including molecular context and transfection efficiency^47^. Notably, HEK-293T cells express the Simian Virus 40 (SV40) large T-antigen, which enhances episomal plasmid replication and supports high levels of transient transgene expression, making this cell line particularly useful for preclinical gene circuit validation^48–52^. To determine the optimal Ang-II triggered AT_1_R-mediated signaling route, we designed a variety of isogenic vectors containing synthetic promoters with specific operator sites or combinations thereof, such as the nuclear factor of activated T cells (NFAT; pMX57, P_NFAT3_-SEAP-pA), cAMP response element (CRE; pCK53, P_CRE_-SEAP-pA), sterol response element (SRE; pGU232, P_SRE6_-SEAP-pA), CRE combined with SRE and NFAT (CSN; pYL1, P_CSN_-SEAP-pA), and nuclear factor kappa light chain enhancer of activated B cells (NFκB; pKR32, P_NFκB_-SEAP-pA) to express reporter genes in an Ang-II-inducible manner (Fig. 1c). Since the broad-spectrum P_CSN_ daisy-chaining the CRE, SRE, and NFAT operator modules showed best-in-class Ang-II-triggered AT_1_R-mediated activation, it was chosen to drive effector gene expression in follow-up experiments (Fig. 1c).

### Characterization and optimization of the antihypertensive synthetic human sensor-effector shunt

**Fig. 2:**
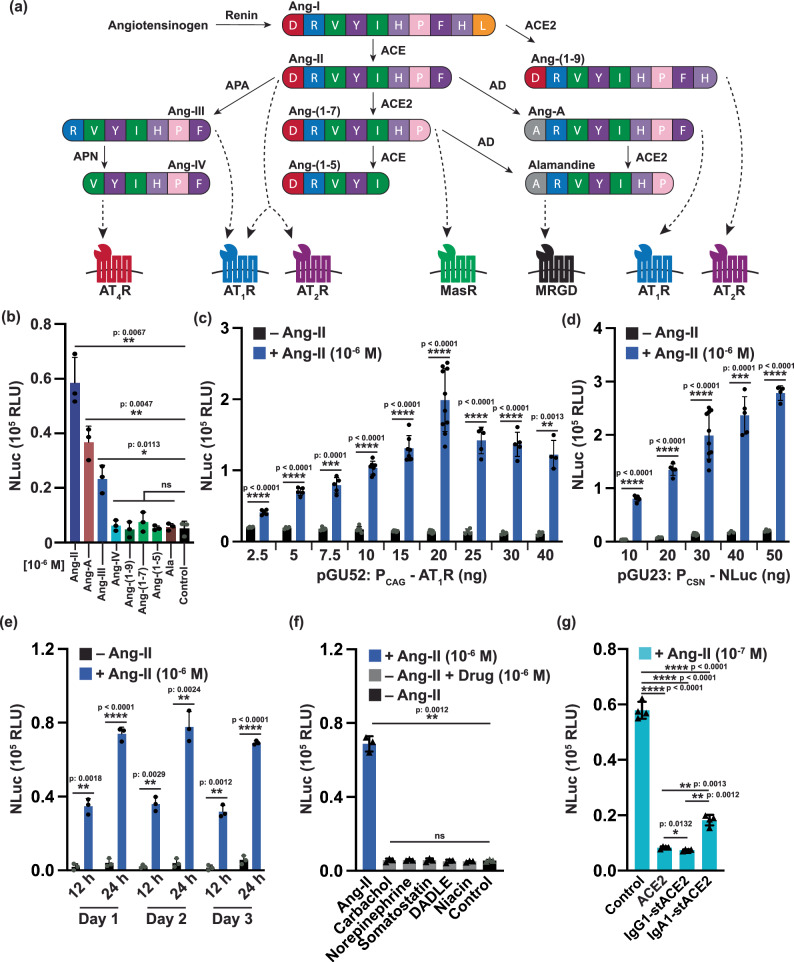
Optimization of the ARCH sensor platform in human cells. **a** Schematic of the renin-angiotensin system (RAS). The RAS contains various angiotensin analogs that interact with different GPCRs. Ang-II is converted by aminopeptidase A (APA) to Ang-III or by ACE2 to Ang-(1–7). Ang-III is further cleaved by aminopeptidase N (APN) to Ang-IV, while Ang-(1–7) is converted to alamandine (Ala) by aspartate decarboxylase (AD). Ang-II and to a lesser extent Ang-III interact with AT_1_R, Ang-II also interacts with AT_2_R, whereas the other angiotensin analogs (Ang-IV, Ang-(1–7), Ala) are ligands to other GPCRs (AT_4_R, MasR, MRGD) within the RAS. **b** Specificity of AT_1_R activation. HEK-293T cells were transfected with ARCH sensor plasmids (pGU23, pGU52) and exposed to 10^−6^ M angiotensin analogs present in the RAS. Only Ang-II and Ang-III were capable of activating the receptor. The angiotensin analogs generated by the ACE2 enzymatic reaction were unable to activate ARCH. Data shown as mean ± SD, *n* = 3 biological replicates. **c** Receptor tunability. Increasing quantities of the transfected receptor vector (pGU52) at fixed reporter (30 ng pGU23) and total plasmid amounts show peak expression and fold-induction levels at the optimal receptor-to-reporter ratio of 2:3. Data shown as mean ± SD, *n* ≤ 4 biological replicates. **d** Reporter tunability. Increasing quantities of the transfected reporter vector (pGU23) at fixed receptor (20 ng pGU52) and total plasmid amounts show a linear increase in reporter gene expression at the cost of decreased fold-induction levels (due to greater leakiness). Data shown as mean ± SD, *n* ≤ 4 biological replicates. **e** Reversibility of AT_1_R activation. HEK-293T cells were transfected with ARCH and cultivated for 72 h. ON (10^−6^ M Ang-II) and OFF states were alternated every 24 h, and measurements were taken every 12 h. Data shown as mean ± SD, *n* = 3 biological replicates. **f** Broader orthogonality of the Ang-II sensor platform. HEK-293T cells co-transfected with pGU23 and pGU52 were exposed to a variety of agonists of GPCRs endogenously expressed in HEK-293T cells. The concentration of each inducer was 10^−6^ M, which is enough to saturate the corresponding GPCRs. None showed any interference with the ARCH sensor platform. Data shown as mean ± SD, *n* = 3 biological replicates. **g** Therapeutic variants. The enzymatic efficiency of different variants of stACE2 was tested in a cell-based functional assay. Data shown as mean ± SD, *n* = 4 biological replicates. In (**b**–**g**): statistical analyses represent unpaired, two-tailed Welch’s *t*-test with a 95% confidence interval, where ns not significant, *p < 0.05, **p < 0.01, ***p < 0.001, ****p < 0.0001. Source data for this figure is available in the Source data file.

To assess the specificity of AT_1_R-mediated cAMP signaling and the downstream P_CSN_ response, HEK-293T cells co-transfected with pGU52 (P_CAG_-hAT1R-pA) and pGU23 (P_CSN_-SS-nLuc-Fc-pA) were stimulated with a panel of angiotensin analogs spanning both the classical and the counter-regulatory arms of the RAS^53^ (Fig. 2a). AT_1_R was fully activated by Ang-II, and to a lesser extent by the Ang-II degradation product Ang-III, whereas the other angiotensin analogs tested did not trigger receptor signaling (Fig. 2b). Aiming to further boost the sensitivity of the sensor-effector shunt, we co-transfected varying amounts of receptor (pGU52) and reporter (pGU23) plasmids. The relative ratio of the system components indeed impacted the overall sensitivity, and optimal performance was observed at a 2:3 receptor-reporter ratio (Fig. 2c, d). The sensor-effector shunt was fully reversible over consecutive cycles of Ang-II-mediated ON and OFF switching (Fig. 2e).

Although AT_1_R taps into endogenous cAMP signaling to address the synthetic P_CSN_ promoter, this signaling pathway was insulated from interference by endogenous GPCRs employing the same signaling cascade. Indeed, neither somatostatin, norepinephrine, carbachol, niacin, nor the synthetic pain-killing opioid DADLE was able to activate cAMP signaling and P_CSN_ when AT_1_R was ectopically overexpressed, despite the presence of the respective cognate GPCRs (SSTR5, ADRA1B, CHRM3, GPR109A, and OPRD1)^54^ (Fig. 2f). This observation is in line with previous reports suggesting that ectopic expression of GPCRs titrates the associated G proteins away from endogenous GPCRs, thereby insulating the rewired synthetic promoter from undesired interference^55^. Transient activation of endogenous signaling cascades by heterologous GPCRs, other cell-surface receptors, or channels does not have any detectable impact on host cells^31,36,55–65^.

Since the AT_1_R-triggered reporter gene expression is probably insufficient to capture the subnanomolar Ang-II levels reached during arterial hypertension, we designed a fully human plug-in feed-forward gene circuit to amplify the Ang-II input and boost the overall output of the device (Supplementary Fig. 2). The booster plug-in consists of a genetic adapter encoding a P_CSN_-driven human sterol regulatory element transcription factor 1 (hSREBF1)^66^ (pGU234, P_CSN_-hSREBF1-pA) that can amplify expression of any transgene and boost the release of desired biopharmaceuticals (Supplementary Fig. 2). However, since we found that we could achieve comparable sensor-effector performance with monoclonal cell lines containing the basic AT_1_R-based input device, we chose to retain the simpler, more compact basic design as it would be preferable for future cell-based therapies.

### Design and validation of the antihypertensive effector device

Soluble angiotensin-converting enzyme 2 (sACE2), which degrades Ang-II, is a central effector of the vasorelaxant axis of the RAS^67^ and was clinically evaluated in Covid-19 studies^40,68,69^. sACE2 should therefore be an excellent antihypertensive effector protein. Since sACE2 is generated by ADAM17-mediated conversion of its membrane-bound form mACE2^70^, we considered direct Ang-II-triggered AT_1_R-mediated P_CSN_-driven expression of a secretion-engineered, stabilized, therapeutic ACE2 variant (stACE2) to establish the autonomous regulator of chronic hypertension (ARCH). We profiled different variants of stACE2 for Ang-II degradation in a cell-based assay using pcDNA3-sACE2(WT)-Fc(IgA1) (immunoglobulin A (IgA) fusion, P_hCMV_-stACE2 (IgA1)-pA), pcDNA3-sACE2(WT)-Fc(IgG1) (immunoglobulin G (IgG) fusion, P_hCMV_-stACE2 (IgG1)-pA), as well as pcDNA3-sACE2-WT(732) (wild-type sACE2, P_hCMV_-sACE2-pA) (Fig. 2g). Since the IgG1-tagged secretion-engineered stACE2-derived proteinase domain combined the most compact 844-amino-acid format with the maximum expression level and optimal angiotensin cleavage activity, we chose it as the therapeutic effector for ARCH. We further validated the enzymatic activity of the therapeutic effector (stACE2 fusion protein) using a fluorometric assay (Supplementary Fig. 3). We additionally confirmed that the recombinant fusion protein is inhibited by MLN4760, a competitive ACE2 inhibitor. It should be noted that beyond catalytic blockade, MLN4760 binding has been reported to induce conformational changes in soluble ACE2 that can modulate non-catalytic interactions and, depending on the assay format, could affect antibody-based detection in a context-dependent manner^71–73^.

### Design of ARCH-transgenic monoclonal human cells (HEK_ARCH_)

**Fig. 3:**
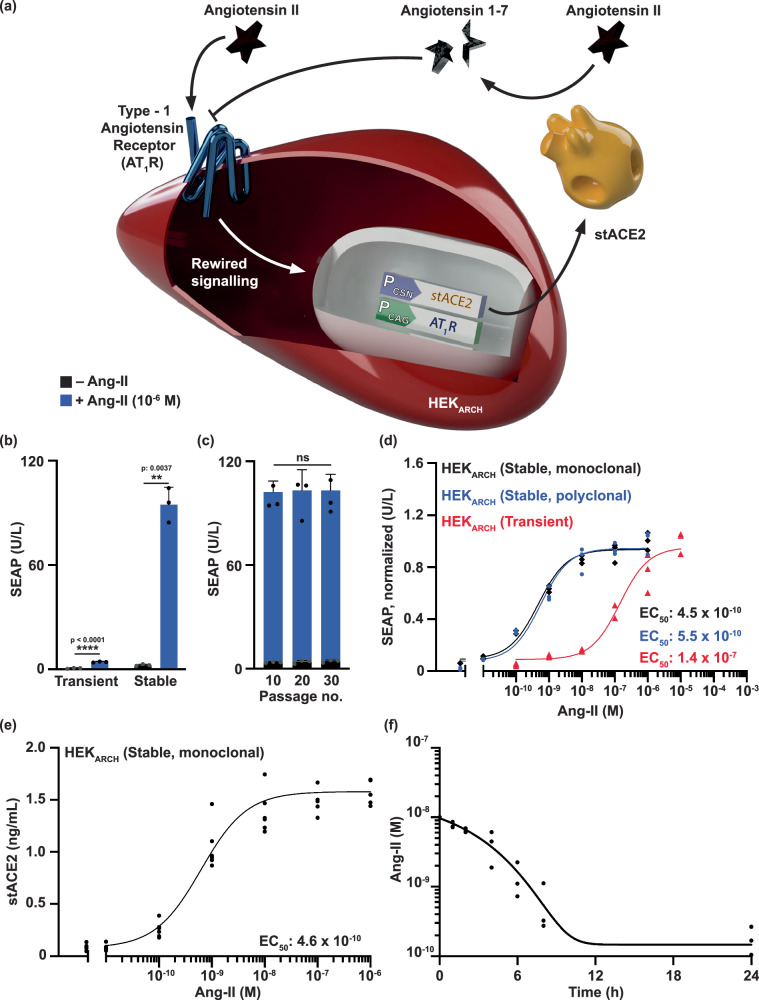
Generation of stable HEK_ARCH_ cells. **a** The binding of Ang-II to AT_1_R triggers activation of the broad-spectrum CSN promoter, which integrates the signaling to initiate therapeutic stACE2 expression. The stACE2 expressed by HEK_ARCH_ cleaves Ang-II into Ang-(1–7), rendering it unable to interact with AT_1_R, and thereby causing (i) the lowering of blood pressure and (ii) the slowing of the therapeutic circuit through feedback inhibition. **b** SEAP reporter expression and fold-induction levels of HEK-293T cells with transient CAG-driven human AT_1_R (pGU52, P_CAG_-hAT_1_R-pA) and CSN-driven SEAP expression (pYL1, P_CSN_-SEAP-pA), as well as of polyclonal cell populations stably expressing human AT_1_R with transient CSN-driven SEAP expression (pYL1, P_CSN_-SEAP-pA). **c** Stability of monoclonal HEK_ARCH_ circuit performance across 30 passages. **d** Ang-II dose-response of a polyclonal cell population with stably integrated P_CAG_-driven human AT_1_R (pGU52) and transient P_CSN_-driven SEAP expression (pYL1), compared with monoclonal HEK_ARCH_ cells with stably integrated P_CAG_-driven human AT_1_R (pGU52) and P_CSN_-driven stACE2 and SEAP (pGU139, P_CSN_-stACE2-IgG1-P2A-SEAP-pA) expression. **e** In vitro Ang-II dose-response characterization of HEK_ARCH_ for stACE2 production was performed using a human ACE2 ELISA. **f** Kinetics of Ang-II depletion mediated by secreted stACE2 from HEK_ARCH_ cells. In (**b**, **c**, **d**, **f**), data shown as mean ± SD, *n* = 3 biological replicates. In (**e**), data shown as mean ± SD, *n* = 6 biological replicates. In (**b**): statistical analyses represent an unpaired, two-tailed Welch’s *t*-test with a 95% confidence interval, where **p < 0.01, ****p < 0.0001. Source data for this figure is available in the Source data file.

Transient co-transfection of ARCH components did not provide sufficient sensitivity to detect Ang-II within the physiologically relevant range. This limitation is consistent with the variability inherent to transient expression, where differences of plasmid copy number and the absence of defined genomic integration can alter transcriptional control parameters, including ligand sensitivity, basal activity (leakiness), inducibility, and absolute expression levels^50,74^. This issue is well recognized across mammalian synthetic gene circuits, where transient co-transfection can introduce substantial cell-to-cell variability^49–52^. We therefore generated stably transgenic HEK-293T cell lines with constitutive expression of human AT_1_R (pGU52, P_CAG_-hAT_1_R-pA) and P_CSN_-driven expression of secretion-engineered stACE2 (pGU139, P_CSN_-stACE2-IgG1-P2A-SEAP-pA) (Fig. 3a, b). To exclude genetic drift or transgene silencing, we evaluated circuit performance across multiple passages and observed stable function over passage numbers aligned with established industrial benchmarks for HEK-293T-derived production cell lines^75,76^ (Fig. 3c). Indeed, Ang-II-triggered dose-response profiles indicated that the polyclonal cell line (EC_50_ = 5.5 × 10^−10^ M) showed a 250-fold higher Ang-II sensitivity than transiently transfected cell cultures (EC_50_ = 1.4 × 10^−7^ M), driving half-maximal effective concentrations (EC_50_) into the clinically relevant subnanomolar range (Fig. 3d). Selecting the stable monoclonal cell line HEK_ARCH_ from the polyclonal cell population further boosted ARCH performance (EC_50_ = 4.5 × 10^−10^ M), affording over 300-fold higher Ang-II sensitivity compared to the basic transient configuration and driving ARCH’s sensitivity well into the clinically relevant sensitivity range (Fig. 3d). Upon stimulation with clinically relevant doses of Ang-II, HEK_ARCH_ produced and secreted stACE2 (Fig. 3e). To quantify the temporal dynamics of stACE2 activity, we monitored circuit output over time and observed a progressive depletion of Ang-II consistent with enzymatic degradation (Fig. 3f).

### Interactions of ARCH with canonical hypertension medications

**Fig. 4:**
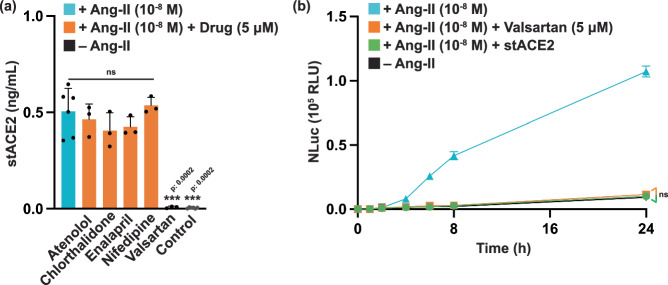
Interaction of ARCH with canonical hypertension medications. **a** One candidate from each of the five main classes of hypertension medication was tested with HEK_ARCH_ having stably integrated CAG-driven human AT_1_R (pGU52) and CSN-driven stACE2 and SEAP (pGU139, P_CSN_-stACE2-IgG1-P2A-SEAP-pA) expression. stACE2 release was blocked only by a working concentration of the clinically licensed AT_1_R antagonist valsartan, which interferes with ARCH’s Ang-II detection and signaling. All analyses are two-tailed *t*-tests versus the Ang-II-only group. **b** The competitive inhibitor valsartan and stACE2 were both able to inhibit the Ang-II/AT_1_R interaction with similar kinetics in vitro. Statistical comparison is column dependence from 2-way ANOVA. In (**a**, **b**), data shown as mean ± SD, *n* = 3 biological replicates. In (**a**, **b**): statistical analyses represent unpaired, two-tailed Welch’s *t*-test with a 95% confidence interval, where ns not significant; ***p < 0.001. Source data for this figure is available in the Source data file.

To profile possible interactions between HEK_ARCH_ and medications targeting its components, we exposed HEK_ARCH_ cells to a variety of antihypertensive medications at physiologically relevant, therapeutic levels (5 μM). HEK_ARCH_-triggered stACE2 release could be blocked by clinically licensed antihypertensive drugs such as valsartan (an AT_1_R antagonist), which interferes with ARCH’s Ang-II detection and signaling. Other blood pressure therapeutics with targets unrelated to ARCH did not interfere with the activity of this antihypertensive hormone shunt within the therapeutic concentration ranges, suggesting that ARCH should be compatible with combination regimes without direct molecular cross-reactivity that would compromise its function (Fig. 4a). Valsartan and ARCH-mediated stACE2 exhibited comparable efficacy in a kinetic assay (Fig. 4b).

### ARCH-based control of experimental hypertension

**Fig. 5:**
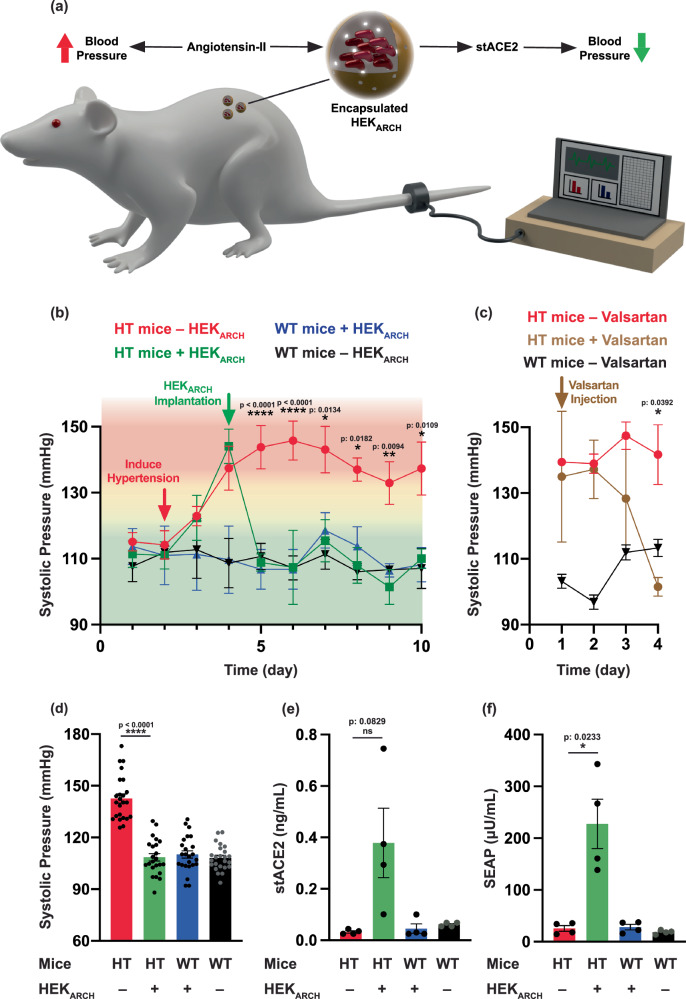
ARCH for angiotensin-dependent hypertension. **a** Schematic of the in vivo experimental model depicting the cell-laden capsule implants as well as the tail-cuff device used to record blood pressure values non-invasively. **b** Blood pressure readings of hypertensive (HT) treated and untreated mice, as well as wild-type (WT) treated and untreated mice, were taken every morning for 10 days. Statistical comparison shown is between the groups [HT mice – HEK_ARCH_] and [HT mice + HEK_ARCH_]. **c** Single, high-dose intraperitoneal valsartan injection (25 mg/kg) lowered the blood pressure of hypertensive mice gradually over 3 days. **d** The disease group treated with ARCH showed a rapid improvement that remained stable for the entire duration of the study. ARCH had no apparent negative effects on the wild-type treated group. Each data point is an individual blood pressure reading starting from day 5. Plasma isolated from day 10 blood samples of each mouse was analyzed with a human ACE2 ELISA kit for stACE2 quantification (**e**) and with a luminescence reporter assay kit for SEAP quantification (**f**). Data shown as mean ± SEM, *n* = 4 mice per group. In (**b**, **c**): statistical analyses represent the linear mixed model described in detail in the methods; in (**d**–**f**): statistical analyses represent an unpaired, two-tailed Welch’s *t*-test with a 95% confidence interval, where *p < 0.05, **p < 0.01, ****p < 0.0001. Source data for this figure is available in the Source data file.

To validate ARCH’s potential to sense Ang-II levels associated with arterial hypertension and produce therapeutic doses of stACE2 to restore and maintain normal blood pressure in an automatic, self-sufficient, and closed-loop manner, we microencapsulated 5 × 10^6^ HEK_ARCH_ into coherent beads of clinically licensed alginate and implanted them subcutaneously in hypertensive mice in an Ang-II infusion model^77–79^ (Fig. 5a). ARCH was able to detect and correct hypertension (>140 mmHg systolic), restore normal blood pressure (<120 mmHg systolic) within 24 h after implantation on day 4, and maintain the systolic pressure within 100 to 120 mmHg for the remainder of the study (Fig. 5b). The diastolic pressure and the mean arterial pressure profiles also supported the therapeutic potential of ARCH (Supplementary Fig. 4). In contrast, a single high-dose treatment of hypertensive mice with the clinically licensed antihypertensive drug valsartan only gradually reduced blood pressure over 72 h, while other hypertension medications failed to reverse the high blood pressure after a single administration, suggesting that ARCH compares favorably with established antihypertensive drugs for the treatment of experimental hypertension (Fig. 5c, Supplementary Fig. 5). No hypotensive excursions were seen either in hypertensive mice receiving the ARCH-based cell therapy or in wild-type animals implanted with HEK_ARCH_ as evidenced by blood pressure profiling and behavioral monitoring (Fig. 5d). Blood level analyses confirmed that only ARCH-treated hypertensive mice showed increased stACE2 in their bloodstream (Fig. 5e), at levels that correlated with those of the co-cistronically expressed reporter protein SEAP (Fig. 5f). Overall, these results represent a proof-of-concept for cell-based therapy to achieve ARCH-mediated closed-loop control of arterial hypertension. In order to verify the host compatibility of the therapeutic strategy, the cell-laden capsules were harvested at 2 weeks post-implantation, together with the surrounding peritoneal lavage. Upon flow-cytometric analysis of the peritoneal lavage and subsequent leukocyte profiling, no abnormality was detected in host immune cell composition^80^ (Supplementary Figs. 6 and 7).

### Closed-loop control of obesity-associated RAS dysregulation

**Fig. 6:**
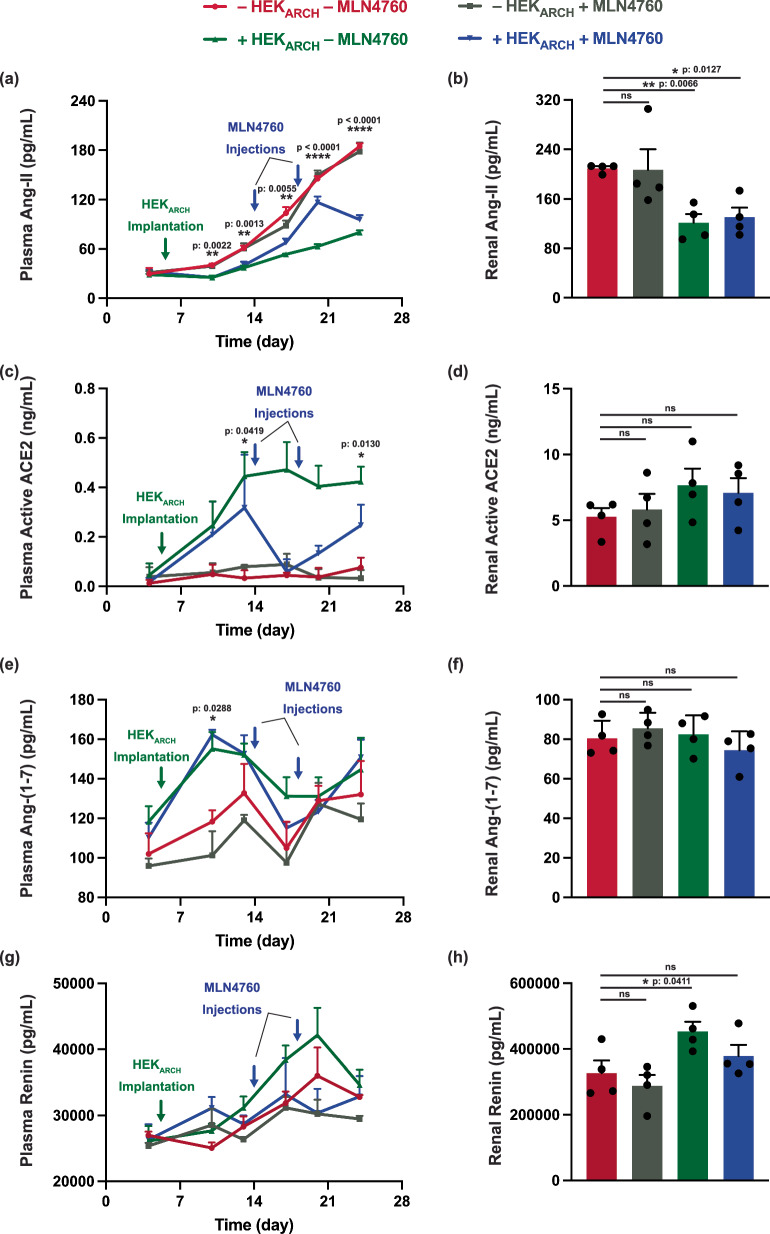
ARCH for RAS dysregulation. **a** Plasma Ang-II levels gradually increased in obese mice, and the increase was attenuated by the HEK_ARCH_ implant. The ACE2-mediated therapeutic effect was transiently blocked by intraperitoneal bolus injections of ACE2 inhibitor (MLN4760, 1 mg/kg). **b** Renal Ang-II levels were significantly reduced in treatment groups. **c** Plasma ACE2, monitored twice weekly, was inversely correlated with plasma Ang-II attenuation. **d** No significant changes were observed in membrane-bound renal ACE2 levels. **e** Plasma Ang-(1–7) levels were transiently elevated in treatment groups following HEK_ARCH_ implantation but subsequently remained stable throughout the experiment. **f** No changes were observed in renal Ang-(1–7). **g**, **h** Plasma and renal renin were elevated in the [+HEK_ARCH_ − MLN4760] group. In (**a**, **c**, **e**, **g**): statistical comparison shown is between the groups [+HEK_ARCH_ − MLN4760] and [−HEK_ARCH_ − MLN4760]. The green arrow indicates the therapeutic cell implantation procedure on day 5, and the blue arrows indicate the intraperitoneal bolus injections of ACE2 inhibitor on day 14 and day 18 (MLN4760, 1 mg/kg). Data shown as mean ± SEM, *n* = 4 mice per group. In (**a**–**h**): statistical analyses represent unpaired, two-tailed Welch’s *t*-test with a 95% confidence interval, where *p < 0.05, **p < 0.01, ****p < 0.0001. Source data for this figure is available in the Source data file.

Elevated Ang-II has been implicated in diverse pathophysiological processes^41–45^. Because obesity is associated with RAS activation, partly driven by adipose tissue as an endocrine organ that upregulates local angiotensin signaling^81^, we used an obesity model to test ARCH functionality under conditions of disease-relevant RAS dysregulation. In high-fat diet-fed mice, both plasma and renal Ang-II levels were elevated, and decreased following ARCH implantation (Fig. 6a, b, Supplementary Fig. 8). Intraperitoneal bolus administration of an ACE2 inhibitor transiently blunted the Ang-II lowering, indicating that stACE2-dependent enzymatic degradation is the primary therapeutic mechanism. Reduced plasma Ang-II levels correlated with increased circulating soluble ACE2 (Fig. 6c), whereas renal ACE2 levels remained unchanged (Fig. 6d). Plasma Ang-(1–7) showed a transient increase in the treatment group but this was not sustained (Fig. 6e), and renal Ang-(1–7) remained unchanged (Fig. 6f). Finally, consistent with reduced AT_1_R signaling, the circulating and renal renin concentrations increased in line with compensatory renin release from juxtaglomerular cells (Fig. 6g, h).

### ARCH responsiveness to human serum

**Fig. 7:**
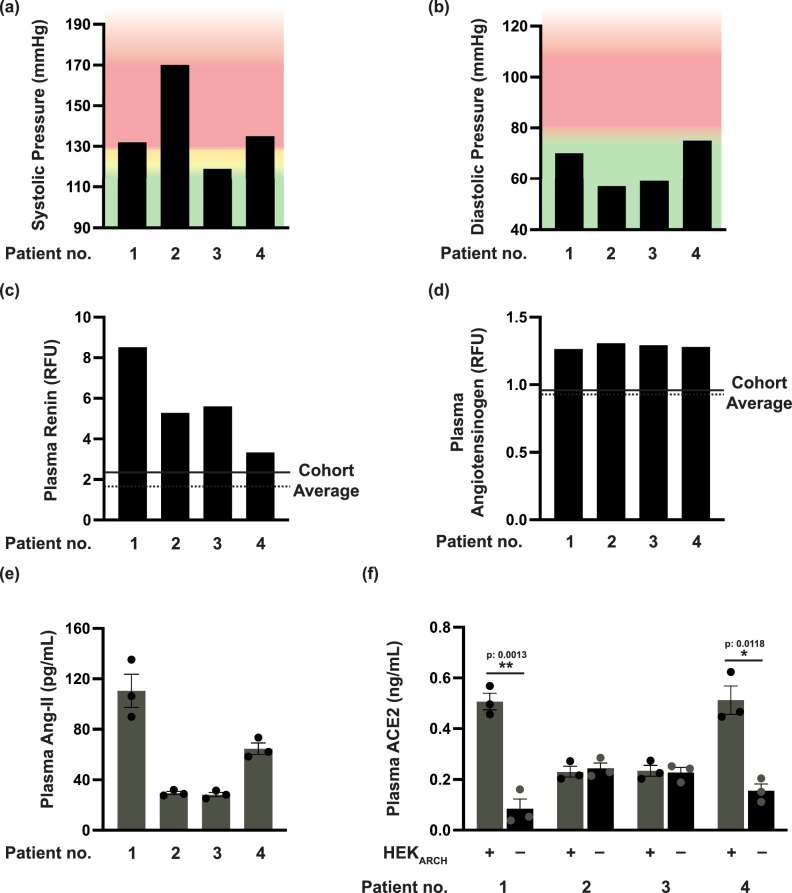
ARCH response to human samples. **a**, **b** Blood pressure of patients who provided serum samples. Elevated systolic and normal diastolic blood pressure measurements indicate isolated systolic hypertension (ISH). **c** Plasma renin levels of the selected patients were two to five times higher than the cohort average. **d** Plasma angiotensin levels of the selected patients were in the 80th percentile. **e** Plasma Ang-II levels. **f** HEK_ARCH_ cultured in human samples achieved significant stACE2 expression for two of the four patients. In (**c**, **d**): solid line indicates the cohort mean, dashed line indicates the cohort median. In (**e**, **f**): statistical analyses represent unpaired, two-tailed Welch’s *t*-test with a 95% confidence interval, where *n* = 3 biological replicates; data shown as mean ± SEM. *p < 0.05, **p < 0.01. Source data for this figure is available in the Source data file.

To probe translational relevance, we cultured HEK_ARCH_ cells in sera from hypertensive individuals. Participants spanned elevated blood pressure (>120 mmHg), stage 1 hypertension (>130 mmHg), and stage 2 hypertension (>140 mmHg) based on systolic values (Fig. 7a) and exhibited isolated systolic hypertension, with diastolic blood pressures in the normal range (<80 mmHg) (Fig. 7b). To enrich for RAS activity, sera were selected from individuals not receiving ACE inhibitors and in the top quintile for renin and angiotensinogen (Fig. 7c, d). Consistent with this stratification, sera containing higher levels of Ang-II activated the AT_1_R/reporter axis and stACE2 expression in HEK_ARCH_ (Fig. 7e, f).

## Discussion

Ever since Paracelsus discovered the drug dose-response correlation, open-loop therapeutic dosing has remained the predominant treatment strategy^82^. However, with the advent of synthetic biology, the engineering of mammalian cells for therapeutic applications has enabled new cell-based dosing paradigms in which in situ production of protein therapeutics by implanted designer cells is triggered by small-molecular compounds such as antibiotics (pristinamycin^83^, erythromycin^84^), vitamins (vitamin H^85^), food additives (xylose^86^, benzoic acid^87^, vanillic acid^88^), and percutaneous cosmetics (phloretin^89^, paraben^90^, spearmint^61^), as well as by inhalable substances (acetaldehyde^84^, acetoin^91^) or physical cues (light^57,92^, temperature^93^, electrical fields^62^). But, although the open-loop design of these trigger-controlled expression systems promises convenient dosing via in situ production, they share the same challenges as classical therapeutics, including side effects (of trigger compounds and therapeutics), interference, and dosing precision, as well as patient compliance and adherence, all of which are critical for the treatment of chronic medical conditions requiring lifelong, precisely-dosed, drug-based interventions^94^. In contrast, cell-based, closed-loop control systems, in which the in situ production of protein pharmaceuticals is directly coordinated by metabolism and biomarkers, can enable automatic, precise, and seamless control of protein therapeutics and have the potential to restore and maintain healthy homeostasis without the need for patient interventions. Such systems still face ongoing challenges, including designer cell longevity, microencapsulation issues such as fibrosis, material leaching, and immunogenicity, non-human orthogonality and interference of the synthetic gene circuits, as well as manufacturing costs and replacement intervals^59,95,96^. Recent advancements, such as the use of antifibrotic alginate formulations, have demonstrated considerable potential in substantially extending the functional lifespan of designer cell implants^94,97^. Nevertheless, inaugural closed-loop cell implants have already illustrated the potential of dynamic homeostasis control for the management of experimental chronic medical conditions^98^, including gouty arthritis^31,32^, obesity^33^ and diabetes^34,36,99,100^. In line with these advances in synthetic biology, complemented by AI-based design of protein therapeutics^101^, we anticipate that the engineering of designer cells for cell-based therapies will gradually shift from xenogeneic gene-switch-controlled, open-loop circuits to all-human, closed-loop systems using native sensor and effector components that are dynamically wired via a synthetic control shunt. Such fully human, closed-loop, therapeutic gene circuits are expected to be safe, robust, and intervention-free, and may even be considered a cure rather than simply a classical therapy managing the symptoms^102^. Such a treatment vision is particularly appealing for chronic disorders associated with long-term complications, side effects, and the need for medication adherence during lifelong therapy^103^.

In this context, hypertension is an attractive target. Various genetic and environmental factors can cause dysregulation of the RAS, leading to arterial hypertension, the most common preventable disease with no permanent cure^104^, and one that can lead to fatal complications and a variety of comorbidities. With the exception of a prototypic personalized stem cell therapy that requires ongoing intravascular infusions^105^, treatment options for arterial hypertension are currently limited to lifelong daily administration of multiple drugs. Thus, there is an urgent need for long-term antihypertensive cell therapies that are precise, robust, and reliable. Because hypertension is etiologically heterogeneous, Ang-II is not a universal biomarker across patient groups. We therefore position ARCH as a closed-loop precision therapy for angiotensin-dependent (RAS-driven) hypertension, in which Ang-II is persistently elevated, and feedback control can autonomously tune therapeutic output in response to inter-individual differences in biomarker dynamics and demand. With this in mind, we designed ARCH, a fully humanized synthetic hormone shunt, in which an ectopic AT_1_R is dynamically activated at subnanomolar concentrations of Ang-II to trigger secretion-engineered stACE2, thereby degrading Ang-II in a feedback-controlled manner. Our findings here show that ARCH rapidly corrected experimental hypertension within a day after implantation of microencapsulated cells and subsequently maintained normal blood pressure without any hypotensive excursion. The therapeutic strategy of ARCH relies on the direct enzymatic degradation of the hypertension agonist, Ang-II, rather than a competitive inhibition of AT_1_R or other hypertension targets. These mechanistically distinct approaches are expected to differ in their efficiency at suppressing AT_1_R signaling in vivo due to pharmacological limitations^106^. In these respects, ARCH indeed compares favorably with classical antihypertensive drugs such as valsartan, which takes several days to lower blood pressure. Consistent with pharmacological RAS blockade, ARCH-mediated attenuation of Ang-II/AT_1_R signaling was accompanied by a compensatory increase in renin, which could, in principle, engage (pro)renin receptor-linked, Ang-II-independent pathways and should be considered in future long-term evaluations.

ARCH capitalizes on stabilized, secretion-engineered stACE2, an Ang-II-degrading key vasorelaxant player in the RAS^67^. The safety of stACE2 is well supported by the widespread use of its wild-type counterpart, sACE2, as a biopharmaceutical for emergency treatment of SARS-CoV-2 infections, which involved twice-daily injections of human recombinant sACE2 at 0.4 mg/kg^107^. Normotensive sACE2 levels of healthy individuals typically reach 0.16 ng/mL^108^. Considering that only 50,000 HEK_ARCH_ cells can produce 1.5 ng of stACE2 per mL, ARCH should, in principle, be scalable for clinical translation. Indeed, ARCH-controlled stACE2 production was effective in restoring and maintaining normal blood pressure in an experimental mouse model of arterial hypertension at levels three orders of magnitude lower than the dose used for anti-SARS-CoV-2 treatment, and only two to three times higher than the normotensive levels. Overall, the comparatively low therapeutic output requirement, together with feedback-limited circuit activity, appears to be consistent with a favorable long-term safety profile, an essential prerequisite for clinical translation. We consider that the development of synthetic, but fully humanized hormone shunts with dynamic physiological closed-loop control to restore pathological dysregulations to homeostatic levels, as seen here with ARCH in our hypertensive mouse model, opens up the exciting possibility of replacing lifelong drug treatments with a once-in-a-lifetime gene or cell-based therapies, amounting to a cure of chronic conditions rather than managing the symptoms.

## Methods

### Plasmid design

The cloning strategies and oligonucleotide sequences used in this study can be found in Supplementary Data 1 in the supplementary information. Briefly, the gene expression vectors used in this study were constructed through digestion with high-fidelity restriction endonucleases (New England Biolabs, Massachusetts, USA) followed by ligation with T4 DNA ligase (New England Biolabs, cat. No. M0202S) with or without PCR amplification using custom oligonucleotides (Merck Life Science, UK) and Q5^Ⓡ^ High-Fidelity DNA polymerase (New England Biolabs, cat. No. M0491S). Quick calf intestinal alkaline phosphatase (CIP, New England Biolabs, cat. No. M0525S) was used to prevent self-ligation of empty backbones. Cloned vectors were transformed into XL10-Gold ultracompetent cells (New England Biolabs, cat. No. C3040H), subsequently purified using a miniprep kit (Zymo Research, Irvine, California, USA, cat. No. D4054), and validated via Sanger sequencing (Microsynth AG, Switzerland).

### Cell culture and transfection

Human embryonic kidney (HEK-293T, ATCC: CRL-3216), COS-7 (ATCC: CRL-1651), MDCK (ATCC: CCL-34), BHK-21 (ATCC: CCL-10), NIH/3T3 (ATCC: CRL-1658), and HepG2 (ATCC: HB-8065) cells were cultured in full medium consisting of Dulbecco’s modified Eagle’s medium (DMEM, high glucose, GlutaMAX^TM^ Supplement, Gibco^TM^, cat. No. 10566016) supplemented with 10% fetal bovine serum (FBS, heat-inactivated at 56 °C for 30 min, Sigma-Aldrich, cat. No. F7524) and 1% penicillin-streptomycin (Gibco^TM^, cat. No. 15070063, final concentration 50 U mL^−1^ penicillin and 50 μg mL^−1^ streptomycin) at 37 °C in a humidified atmosphere containing 5% CO_2_. Immortalized human mesenchymal stem cells (hMSC-TERT) were a gift from Moustapha Kassem (University Hospital of Aarhus and University Hospital of Odense, Denmark) and were treated in the same way. Cells were trypsinized at 90-95% confluency with 0.05% trypsin-EDTA (Life Technologies, California, USA; cat. No. 25300-054) for 2 min at 37 °C, then the trypsin was diluted 1:5 in full medium, and the mixture was centrifuged at 250 × *g* for 1 min. The supernatant was discarded, and the cell pellet was resuspended in full medium. The number of cells in suspension was determined using a CellDrop brightfield cell counter (DeNovix, Delaware, USA). For passaging, cells were seeded in culture dishes in the appropriate volume of culture medium at 15,000 cell cm^−1^ surface area. For fast-forward transfection for in vitro experiments, cells were seeded in 96-well plates at 30,000 cells 100 μL^−1^ per well, and cells in each well were immediately transfected with 150 ng total plasmid DNA:600 ng polyethyleneimine (PEI; Polysciences Inc.) mixture in 50 μL FBS-free and antibiotic-free medium for 16 h.

### Monoclonal cell line generation

For HEK_ARCH_ stable cell generation, HEK-293T cells were seeded in 6-well plates at 600,000 cells 2 mL^−1^ per well, and cells in each well were immediately transfected with 2500 ng plasmid DNA (500 ng pGU52 + 2000 ng pGU139):250 ng Sleeping Beauty transposase vector (pSB100X):12,000 ng PEI mixture in 1 mL FBS-free and antibiotic-free medium for 16 h. Stable cell selection was carried out for 10 days with blasticidin (Invitrogen, 5 μg mL^−1^) and puromycin (Invitrogen, 2.5 μg mL^−1^). Next, the dual antibiotic-resistant polyclonal population underwent FACS-mediated single-cell sorting for dual Ypet and mRuby fluorescence. Of the 96 clones picked, 25 surviving clones were screened for Ang-II sensitivity and protein expression. Following clonal expansion, the best-performing clone (HEK_ARCH_) was used for further experiments.

### Inducers

Ang-II (cat. No. 05-23-0101), Ang-III (cat. No. 10385), Ang-IV (cat. No. A0230), Ang-(1–7) (cat. No. A9202), alamandine (cat. No. SML1374); nifedipine (cat. No. N7634), valsartan (cat. No. SML0142), chlorthalidone (cat. No. SML0591), enalapril (cat. No. E6888), atenolol (cat. No. A7655), norepinephrine (cat. No. A7257), [D-Ala^2^, D-Leu^5^]enkephalin (DADLE, cat. No. E7131), carbachol (cat. No. C4382), somatostatin (cat. No. S9129), and niacin (cat. No. N4126) were purchased from Sigma-Aldrich. Ang-(1–5) (cat. No. HY-P1839A) was purchased from MedChemExpress. Ang-A (cat. No. P001208) and Ang-(1–9) (cat. No. P001207) were purchased from Aapptec. MLN4760 (cat. No. 3345) was purchased from Tocris. Stock solutions were prepared in either Dulbecco’s phosphate-buffered saline (DPBS, ThermoFisher Scientific, cat. No. J67670.K2) whenever possible or dimethyl sulfoxide (DMSO, Sigma-Aldrich, cat. No. D8418) if necessary. Stock solutions were aliquoted to avoid multiple freeze-thaw cycles and diluted to the required working concentrations before each experiment.

### Fluorescence microscopy

For fluorescence imaging, cells expressing fluorescent fusion proteins were seeded in black 96-well plates with a transparent flat bottom (Greiner Bio-One, cat. No. 655096). YFP fluorescence was acquired with 520 nm laser excitation 560 nm emission using a Nikon WF3 + X-light SD confocal microscope.

### Flow cytometry

Anti-mouse CD16/32 (93, 101302, 1:200 dilution), anti-mouse CD4 (RM4-5, 100527, 1:500 dilution), anti-mouse CD8β (YTS256.7.7, 126617 and 126633, 1:500 dilution), anti-mouse F4/80 (BM8, 123107, 1:500 dilution), anti-mouse CD25 (PC61, 102011, 1:500 dilution), anti-mouse/human CD44 (IM7, 103006, 1:500 dilution), anti-mouse CX_3_CR1 (SA011F11, 149009, 1:500 dilution), anti-mouse PD-1 (29F.1A12, 135205, 1:500 dilution), anti-mouse CD11c (N418, 117308, 1:500 dilution), and anti-mouse CD80 (16-10A1, 104734, 1:500 dilution) antibodies were purchased from BioLegend. For surface marker staining, cells harvested from peritoneal lavage were collected in U-bottom 96-well plates, blocked with anti-mouse CD16/32 antibody, and incubated with the indicated antibodies at 4 °C for 20 min, followed by live/dead staining with 4,6-diamidino-2-phenylindole (DAPI, Sigma-Aldrich, D9542, final concentration: 0.1 μg ml^−1^). Cells were washed and resuspended in FACS buffer containing 0.2% w/v bovine serum albumin (BSA, Sigma-Aldrich, A3059) and 2 mM EDTA (Invitrogen, AM9260G) in DPBS for flow-cytometric analyses. Flow cytometry was performed using a CytoFLEX S Flow Cytometer with CytExpert Software v2.1 (Beckman Coulter) at the Single Cell Facility of D-BSSE. Data analyses were performed using FlowJo 10.9 (Tree Star). Gate margins were determined by isotype controls and fluorescence-minus-one controls.

### Cell encapsulation

Subcutaneous implants for in vivo experiments were produced by encapsulating HEK_ARCH_ as well as native HEK-293T in alginate-poly-(l-lysine)-alginate (400 µm beads; 200 cells/capsule) using an Inotech Encapsulator Research Unit IE-50R (Buchi Labortechnik AG) according to the manufacturer’s protocol, using a 200 µm nozzle with a vibration frequency of 1024 Hz and 1200 V for bead dispersion, with a 20 mL syringe operated at a flow rate of 500 units. The cell-laden capsules were kept in full culture medium overnight at 37 °C following the encapsulation procedure. Prior to subcutaneous administration, the capsules were washed three times with serum- and antibiotic-free medium.

### Reporter assays

For in vitro secreted embryonic alkaline phosphatase (SEAP) analysis, 20 µL cell supernatant from cells expressing SEAP was diluted in 80 µL water and heat-inactivated at 65 °C for 35 min with continuous shaking at 300 rpm. The assay mixture containing 80 µL buffer (20 mM homoarginine, 1 mM MgCl_2_, 21% (v/v) diethanolamine) and 20 µL substrate (20 mM pNPP, Acros Organics BVBA) was added immediately prior to absorbance measurement at 405 nm using a Tecan M1000 plate reader (TECAN AG, Switzerland). For SEAP analysis of in vivo plasma samples, a luminescence reporter gene assay kit (Abcam, cat. No. ab133077) was used according to the manufacturer’s protocol. For in vitro nanoluciferase analysis, the Nano-Glo Luciferase Assay System (Promega, Wisconsin, USA, cat. No. N1110) was used according to the manufacturer’s protocol.

### RAS measurements

(i) *Cell-based stACE2 assay*. Two transfections were carried out in parallel. For the first transfection, 50 ng of vectors encoding for constitutive expression of different variants of the therapeutic stACE2 (pcDNA3-sACE2(WT)-Fc(IgA1) (immunoglobulin A (IgA) fusion, P_hCMV_-stACE2 (IgA1)-pA), pcDNA3-sACE2(WT)-Fc(IgG1) (immunoglobulin G (IgG) fusion, P_hCMV_-stACE2 (IgA1)-pA), as well as pcDNA3-sACE2-WT(732) (wild-type sACE2, P_hCMV_-sACE2-pA)) was transfected into native HEK-293T with 100 ng pcDNA3.1 (+) filler plasmid. A constitutive eGFP expression vector (pFOX12, P_hCMV_-eGFP-pA) was used as a control. For the second transfection, plasmids encoding CAG-driven AT_1_R expression (pGU52, 10 ng) and CSN-driven nLuc reporter expression (pGU23, 20 ng) were transfected in native HEK-293T cells with 120 ng pcDNA3.1 (+) filler plasmid. The next day, the medium in the second plate was replaced with a mixture of 75 µL supernatant from the first plate and 75 µL of 2 × 10^−6^ M Ang-II. The mixture was incubated at 37 °C for 5 min prior to this. Luminescence was quantified 24 h after this medium change. (ii) *stACE2 quantification*. A human ACE2 ELISA Kit (ThermoFisher Scientific, cat. no. EH489RB) was used according to the manufacturer’s protocols to quantify stACE2 for both in vitro cell culture supernatants and in vivo plasma samples. Cell culture supernatants were diluted with assay diluent 1:1, and plasma samples were diluted with assay diluent 1:2. (iii) *Assay of stACE2-mediated Ang-II degradation kinetics*. HEK_ARCH_ cells were stimulated with Ang-II (1 × 10^−6^ M) to trigger stACE2 secretion, and culture supernatants were collected at 1, 2, 4, 6, 8, and 24 h post-stimulation. In parallel, native HEK-293T cells were transiently transfected with plasmids encoding P_CAG_-driven AT_1_R (pGU52, 10 ng) and P_CSN_-driven nLuc reporter (pGU23, 20 ng), supplemented with 120 ng pcDNA3.1 (+) filler plasmid. For the bioassay, transfected HEK-293T cells were induced with 75 µL HEK_ARCH_ supernatant at each time point and 75 µL of 2 × 10^−8^ M Ang-II. The mixture was incubated at 37 °C for 5 min prior to this. Luminescence was quantified 24 h after this medium change. (iv) *Ang-II quantification*. HEK_ARCH_ cells were transiently transfected with a P_CSN_-driven NanoLuc reporter plasmid (pGU23; 120 ng per well). After transfection, cells were incubated for 24 h with in vitro samples (cell-culture supernatants) or in vivo samples (plasma or serum). NanoLuc luminescence was measured, and Ang-II concentrations were determined by interpolation from an Ang-II standard curve assayed in parallel. For each sample matrix (supernatant, plasma, serum), the corresponding matrix-only control (no Ang-II) was included, and signals were background-corrected. (v) *Ang-(1–7) quantification*. Ang-(1–7) concentrations in in vivo samples were quantified using an Ang-(1–7) ELISA kit (Abbexa; cat. no. abx251960). (vi) *Renin quantification*. Renin levels in in vivo samples were quantified using a mouse renin 1 ELISA kit (RayBiotech; cat. no. ELM-Renin1-1). (vii) *ACE2 activity assay*. ACE2 enzymatic activity was measured using a fluorometric ACE2 activity assay kit (Abcam; cat. no. ab273297).

### Animal experiments

Experiments involving the Ang-II-dependent hypertension model were performed according to the directive of the European Community Council (2010/63/EU), approved by the French Republic (project No. DR2018-40v5 and APAFIS #16753), and carried out by Ghislaine Charpin-El Hamri (license No. 69266309) at the Institut Universitaire de Technologie of the Université Claude Bernard Lyon 1, F-696226, Villeurbanne Cedex, France. Male C57BL/6J mice, aged 12 weeks, weighing 24–26 g, were purchased from Janvier Labs (53940 Le Genest-Saint-Isle, France) and acclimatized for 1 week (*n* = 4). The standard hypertensive mouse model was generated on day 2 through cranial dorsal subcutaneous implantation of micro-osmotic pumps (ALZET, Model 1002, DURECT Corporation, California, USA) loaded with 100 μL aqueous solution of 2.88 mM Ang-II (Calbiochem, cat. no. 05-23-0101, Germany) to achieve 500 ng kg^−1^ min^−1^ infusion via osmosis. Therapeutic cell capsules were injected subcutaneously (1 mL, 5 × 10^6^ cells per mouse) on day 4 in the caudal region. Blood pressure measurements were taken every day in the morning using the non-invasive BP-2000 tail-cuff machine (Bioseb, France). Mice were trained and acclimatized to tail-cuff plethysmography. During measurements, animals were maintained at 37 °C using the instrument’s temperature controller and restrained using appropriately sized holders according to the manufacturer’s guidelines to ensure reliable recordings. On day 10, blood samples were harvested for further analysis. For drug injections, the maximal human doses were converted to corresponding mouse doses, taking account of both weight and surface area as described^109^. For leukocyte profiling, encapsulated cells were injected intraperitoneally (1 mL, 5 × 10^6^ cells per mouse) and recovered together with the surrounding intraperitoneal lavage at 2 weeks post-injection. For RAS profiling, obesity-model experiments were conducted according to the Swiss animal welfare legislation (art. 18, 141, 30), were approved by the Veterinary Office of Canton Basel-Stadt (GenoMet; national No.: 35863, cantonal No.: 3200), and were carried out by Yu-Qing Xie at the ETH Zurich (D-BSSE, CH-4056, Basel, Switzerland). Male B6.V-Lep^ob^/JRj (ob/ob) mice (4 weeks old) were obtained from Janvier Labs (France), acclimatized for 1 week (*n* = 4), and fed a high-fat diet (SAFE-Lab, Augy, France; cat. No. U8956) unless otherwise indicated. Therapeutic cell capsules were administered intraperitoneally (1 mL, 5 × 10^6^ cells per mouse), and blood was sampled twice weekly for subsequent analyses. All mice were continuously monitored (appearance, breathing, eyes, behavior, posture, locomotion, seizure, and weight) throughout the study to ensure their well-being and accurate data collection. All mice were housed in the joint animal facility of Biozentrum University of Basel & D-BSSE ETHZ and were kept in individually ventilated cages, at 19–23 °C, with 45–65% humidity and with a 12-h dark/light cycle.

### Human samples

All participants provided written informed consent, and the study was approved by the Ethics Commission Northwest- and Central Switzerland (IRB00003565). Serum was obtained from hypertensive patients with high angiotensinogen and renin levels (top quintile for each), who were not receiving ACE-inhibitor therapy. Angiotensinogen and renin were measured with a SOMAscan v4 (SomaLogic, Colorado, USA).

### Statistical analysis

The unpaired, two-tailed Welch’s *t*-test was applied with a 95% confidence interval with a minimum of three replicates per sample using GraphPad Prism 8 (v 9.2.0, GraphPad Software Inc.) and Microsoft Excel (v 16.51, Microsoft Inc.). Error bars represent mean ± SD for in vitro measurements and mean ± SEM for in vivo measurements (*p <  0.05, **p <  0.01, ***p <  0.001, ****p <  0.0001). For the in vivo time course experiments, a linear mixed model (*m*) was used in R to describe the average responses for each group on each day; $m={{\mathrm{lmer}}}({{\mathrm{bp}}} \sim {{\mathrm{ht}}}\times {{\mathrm{treat}}}\times {{\mathrm{day}}}+(1{{\mathrm{|mouse}}}),{{\mathrm{data}}}={{{\rm{d}}}})$, where lmer() is the linear mixed-effects regression function, in this case modeling blood pressure (bp) looking at two-by-two groups; hypertensive (ht) versus non-hypertensive (nonht) and treatment (treat) versus control (ctrl). A minimum of three replicates were used for each condition for in vitro experiments. For in vivo experiments, a minimum of four were used. In accordance with European legislation (Directive 2010/63/EC) and the corresponding national regulations governing animal experimentation in the EU, the UK, and Switzerland, animal experiments are conducted under the principles of replacement, reduction, and refinement (3R), with the aim of using the minimum number of animals required to achieve the scientific objective. No data were excluded from the analyses. All in vitro data were successfully reproduced at least twice with proper controls to ensure that no external factors (cell viability, transfection efficiency, etc.) affect data accuracy. For in vivo experiments, mice allocation into different groups was randomized. Investigators were blinded to group allocation during data collection and analysis.

### Reporting summary

Further information on research design is available in the Nature Portfolio Reporting Summary linked to this article.

### Ethics

This study complies with all relevant ethical regulations and all experiments comply with the European Community Council (2010/63/EU), approved by the French Republic (project No. DR2018-40v5 and APAFIS #16753) and Swiss animal welfare legislation (art. 18, 141, 30), approved by the Veterinary Office of Canton Basel-Stadt (GenoMet; national No.: 35863, cantonal No.: 3200).

## Supplementary information

Supplementary Information

Peer Review File

Description of Additional Supplementary Files

Supplementary Data 1

Supplementary Data 2

Reporting Summary

## Source data

Source data

## Data Availability

The authors declare that all data supporting the findings of this study are available herein. Source data are provided with this paper. The data generated in this study are provided in the Supplementary Information/Source data file. Annotated plasmid sequences are provided in Supplementary Data 2. All original plasmids used in this study are available from the authors. Source data are provided with this paper.
