# Supplementary material for: A synthetic angiotensin II/ACE2-based hormone shunt controlling experimental hypertension
Source: Nat Commun. 2026 Apr 11;17:5116. doi: 10.1038/s41467-026-71796-z (PMC13247273; doi:10.1038/s41467-026-71796-z)
Supplement: Supplementary file 1 — Supplementary Information [file 41467_2026_71796_MOESM1_ESM.pdf]

Supplementary Information

A synthetic angiotensin II/ACE2-based hormone shunt controlling experimental hypertension

Gokberk Unal[1], Maysam Mansouri[1], Yu-Qing Xie[1], Christian Mueller[2], Ghislaine Charpin-El Hamri[1,3], Martin Fussenegger[1,4,*]

[1]*Department of Biosystems Science and Engineering, ETH Zurich, Schanzenstrasse 48, CH-4056 Basel, Switzerland*

[2]*Department of Cardiology, University Hospital Basel, Petersgraben 4, CH-4031 Basel, Switzerland*
[3]*Département Génie Biologique, Institut Universitaire de Technologie, F-69622 Villeurbanne Cedex, France*
[4]*Faculty of Science, University of Basel, Schanzenstrasse 48, CH-4056, Basel, Switzerland*

[*]*Correspondence: fussenegger@bsse.ethz.ch*

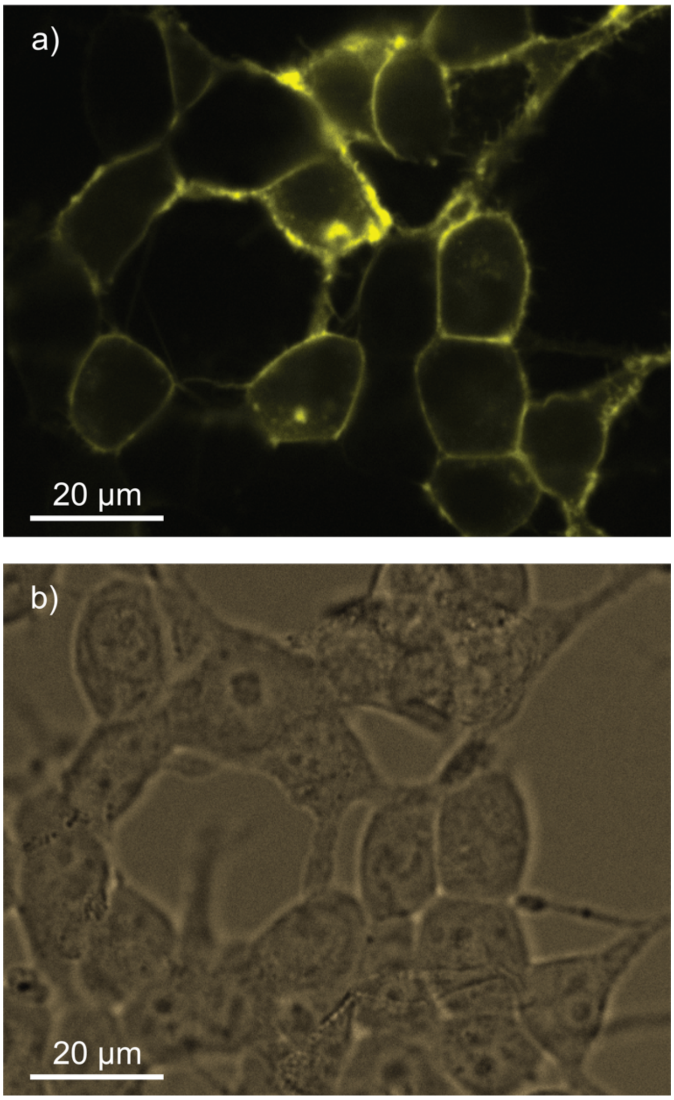

Supplementary Figure 1 | Localization of ectopic hAT_1R to the plasma membrane of human cells. Confocal fluorescence **(a)** and bright-field **(b)** micrographs of HEK-293T cells transfected with pCXN2 encoding hAT_1R fused to yellow fluorescent protein (YFP).

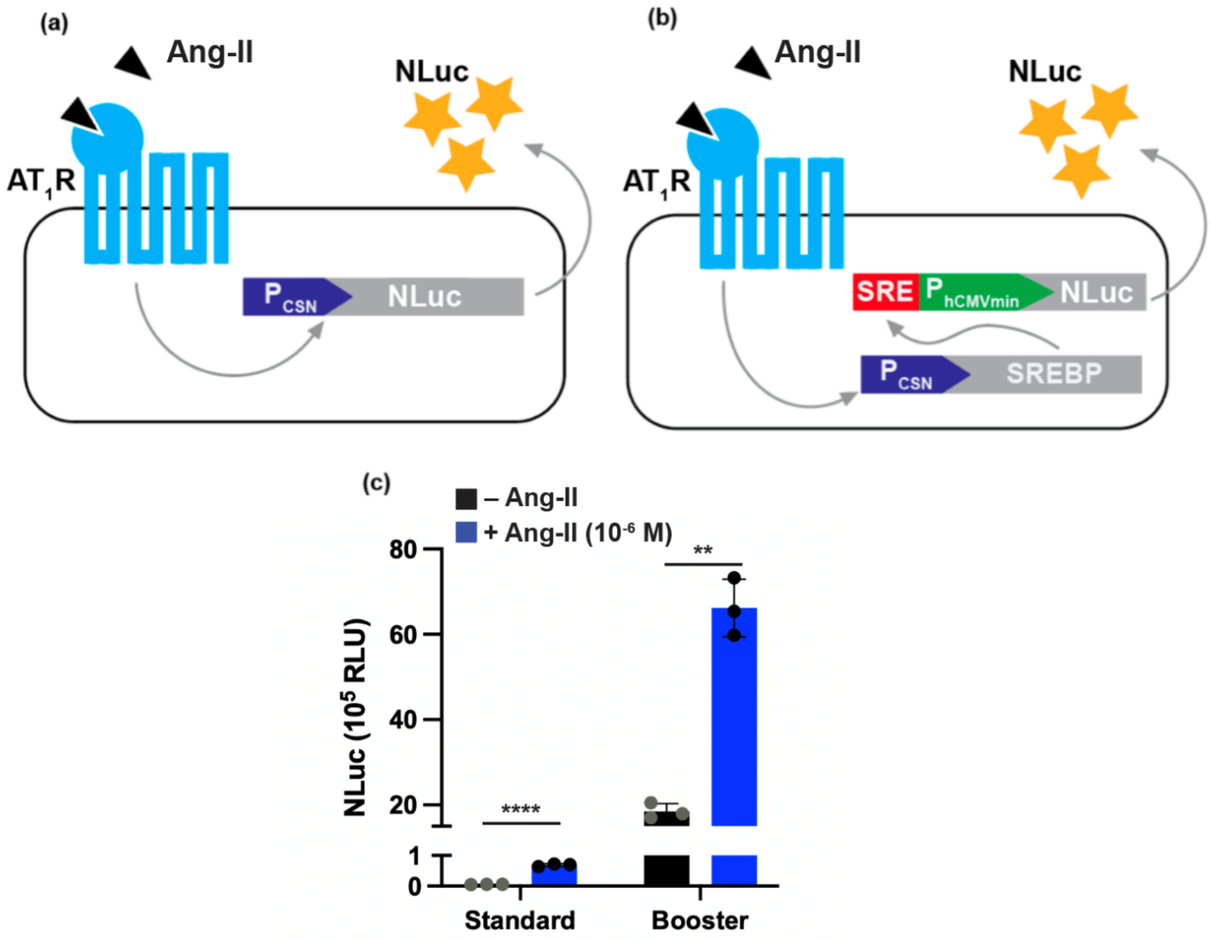

Supplementary Figure 2 | Sensor booster design and performance. (a) Basic Ang-II sensor platform in which Ang-II activates AT_1R, triggering P_{CSN}-driven nLuc expression. **(b)** A fully human plug-in feed-forward gene circuit amplifying the Ang-II input and boosting the overall output of the device. The booster design consists of an intermediate amplification cycle plugged in between AT_1R signaling and nLuc expression and includes AT_1R-activated, P_{CSN}-driven expression of the human transcription factor 1 hSREBF1 (human sterol regulatory element binding transcription factor 1, pGU228) followed by hSREBF1-triggered P_{SRE}-driven expression of nLuc (pGU234). **(c)** Comparative performance analysis of basic and booster sensor variants in HEK-293T cells exposed to Ang-II. Data shown as mean ± SD, n = 3 biological replicates. In panel c: statistical analyses represent unpaired, two-tailed Welch's t-test with a 95% confidence interval, where ****p <0.001, *****p <0.0001*. Source data for this figure is available in the Source Data file.

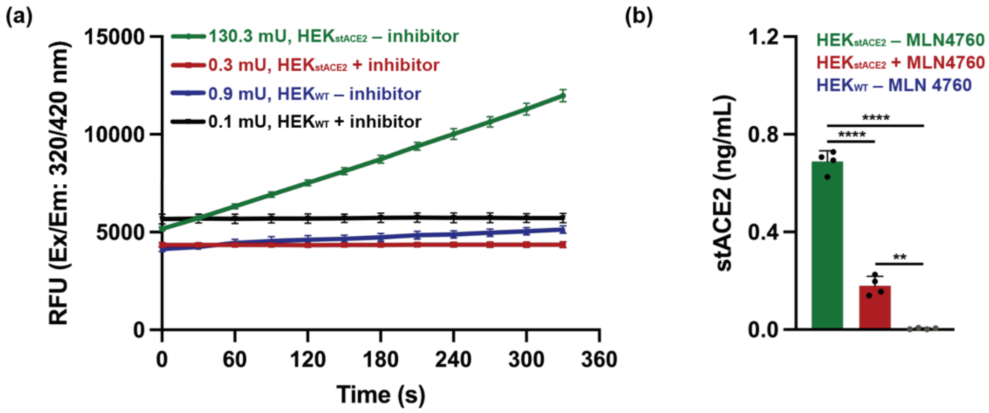

Supplementary Figure 3 | Recombinant ACE2 activity and ACE2 inhibition profile. (**a**) Recombinant ACE2 activity was further verified with a fluorometric ACE2 activity assay. Unit definition: One unit (U) of ACE2 activity is the amount of enzyme that catalyzes the release of 1 nmol of 7-methoxycoumarin fluorochrome per minute from the ACE2 substrate. (**b**) MLN4760 binding to soluble ACE2 has been reported to induce conformational changes that, depending on the assay format, can reduce antibody-based detection by ELISA Data shown as mean ± SD, n = 4 biological replicates. In panel b: statistical analyses represent unpaired, two-tailed Welch's t-test with a 95% confidence interval, where ***p <0.01, ****p <0.0001*. Source data for this figure is available in the Source Data file.

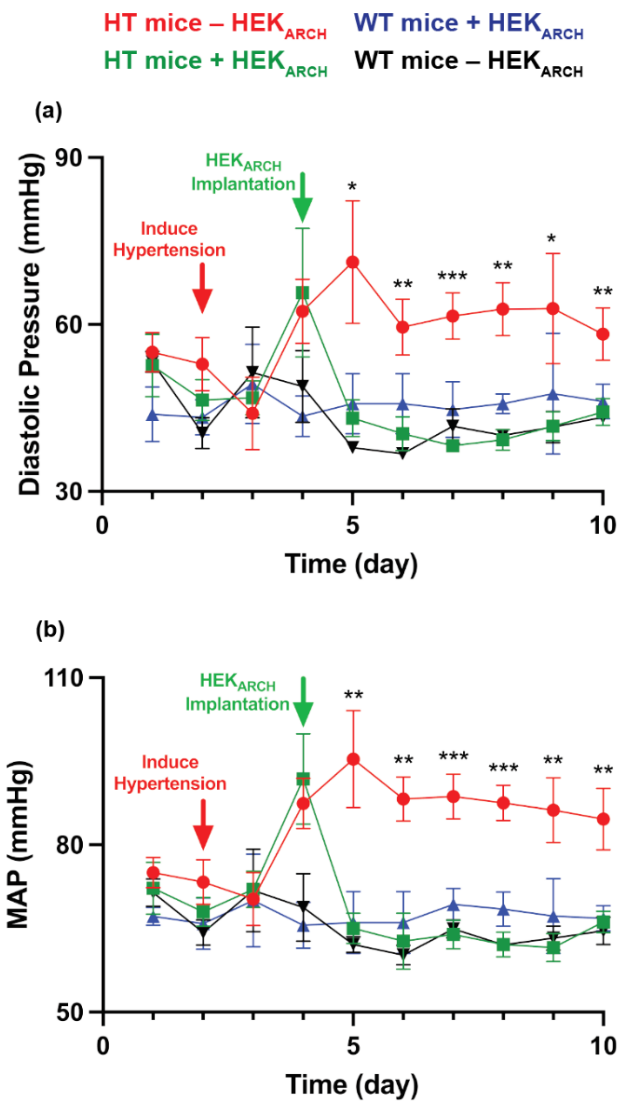

Supplementary Figure 4 | Supplemental data for the Ang-II infusion model. The diastolic pressure **(a)** and the mean arterial pressure (MAP) **(b)** profiles also showcase the restoration of homeostatic blood pressure levels by ARCH in Ang-II-infused, hypertensive (HT) mice. Statistical comparison is between the HT mice – HEK_{ARCH} and HT mice + HEK_{ARCH} groups. Data shown as mean ± SEM, n = 4 mice. In panels a-b: statistical analyses represent the linear mixed model described in detail in the methods, where **p* <0.05, ***p* <0.01, ****p* <0.001. Source data for this figure is available in the Source Data file.

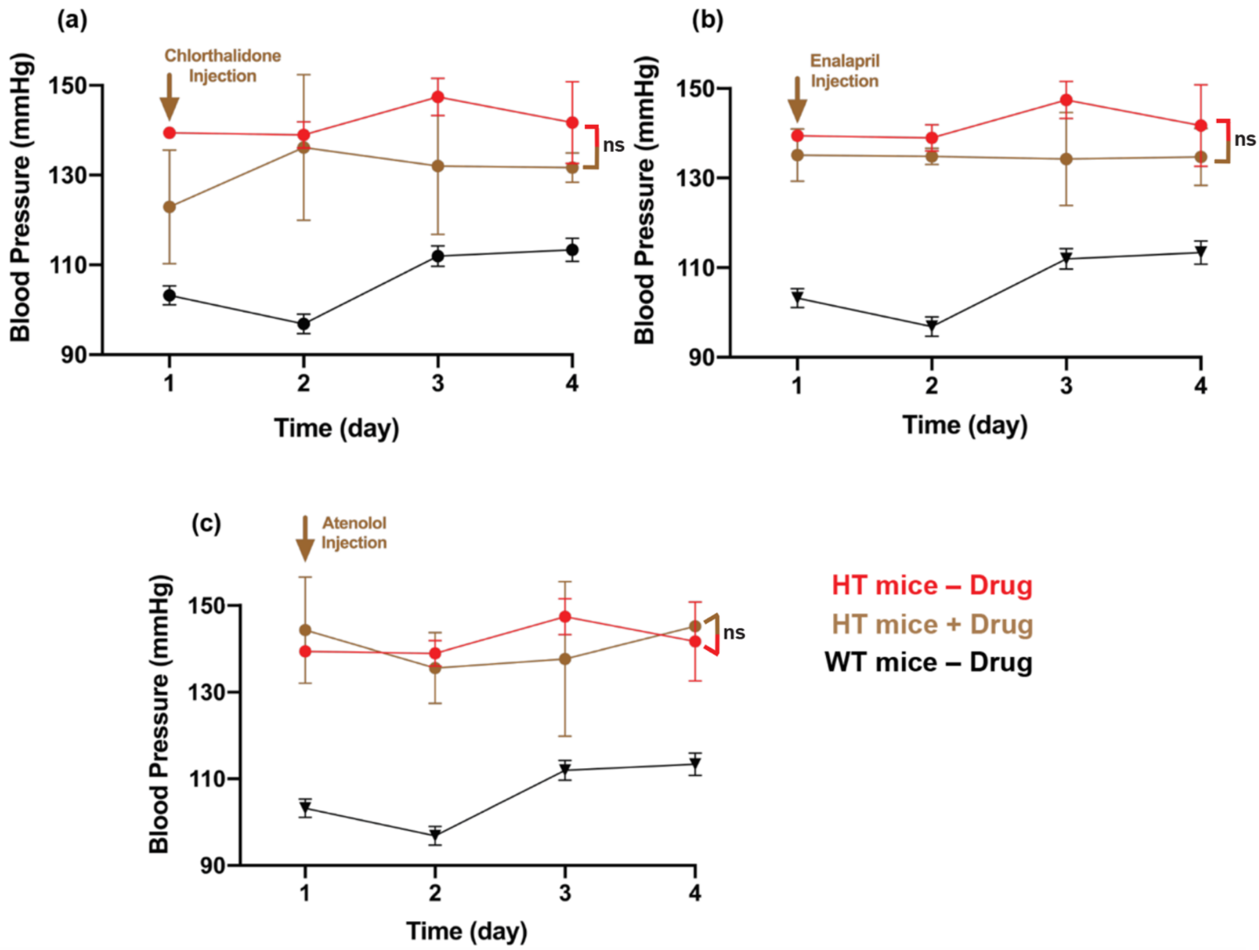

Supplementary Figure 5 | Effects of other antihypertensive agents. Unlike valsartan (Fig. 4c), other canonical antihypertensive drugs showed no impact on chronic experimental hypertension after a single intraperitoneal injection of a dose corresponding to that used in human therapy. **(a)** Chlorthalidone (7.5 mg/kg). **(b)** Enalapril (3 mg/kg). **(c)** Atenolol (15 mg/kg). Data shown as mean ± SEM, n = 4 mice. Statistical comparison is column dependence from 2-way ANOVA. In panels a-c: ns not significant.

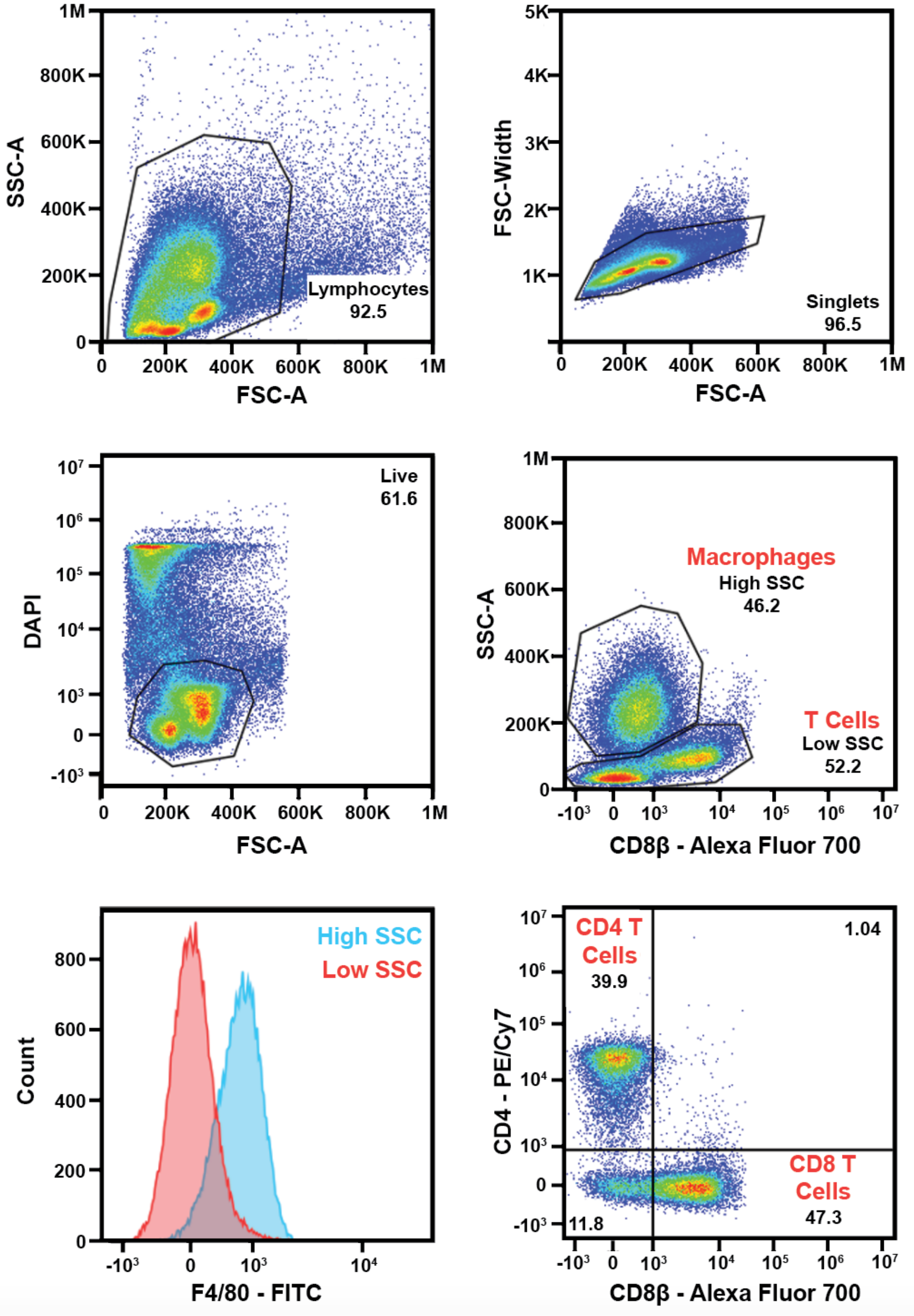

Supplementary Figure 6 | Leukocyte profiling. The cell-laden capsules were harvested two weeks post-implantation together with the surrounding peritoneal lavage. The peritoneal lavage was analyzed by flow cytometry to obtain the leukocyte profile and to check for any abnormality in the immune cell composition.

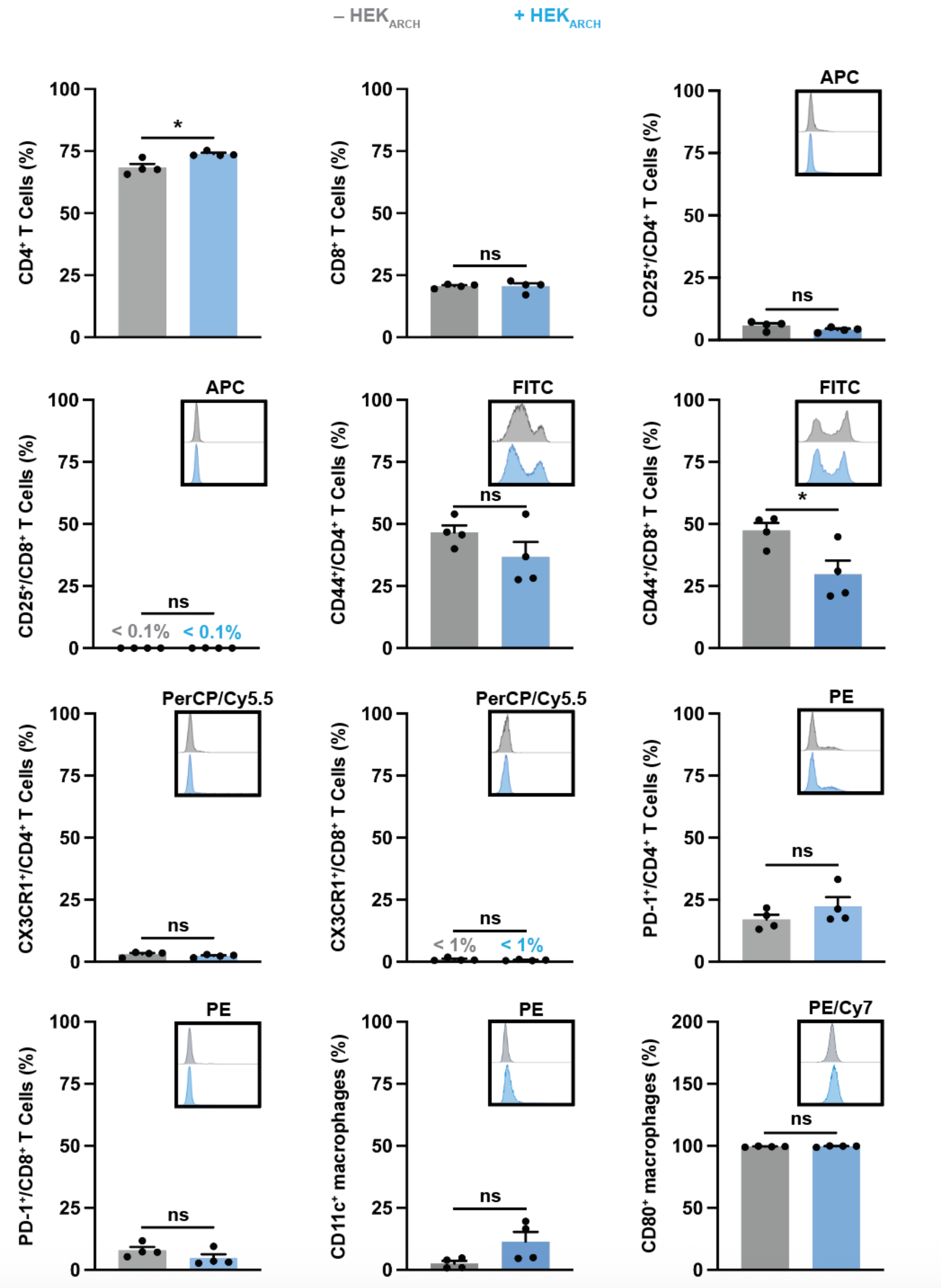

Supplementary Figure 7 | Leukocyte profiling - continued. Intraperitoneal lavage was collected from mice with or without HEK_{ARCH} implants and analyzed by flow cytometry to profile leukocyte populations and assess potential alterations in immune cell composition. Data shown as mean ± SEM, n = 4. *$p < 0.05$.

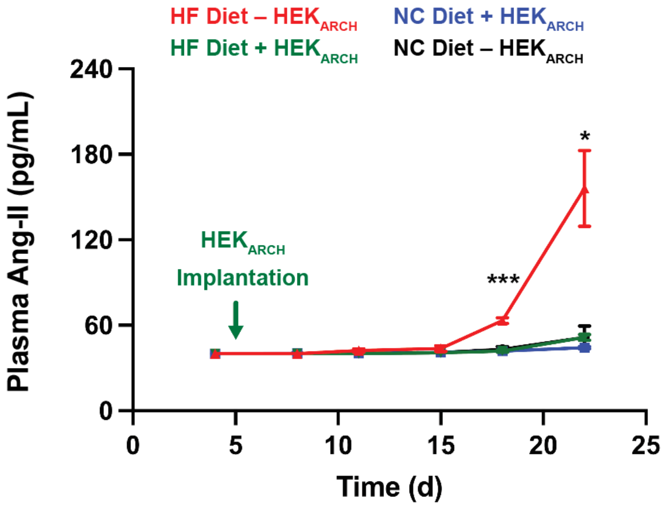

Supplementary Figure 8 | ARCH for obesity-associated RAS dysregulation with normal chow control. Plasma Ang II levels increased over time in high-fat (HF) diet-fed mice and were attenuated by HEK_{ARCH} implantation. Normal chow (NC) diet-fed mice did not display significant elevation of circulating Ang II levels. Statistical comparisons shown are between the groups HF diet - HEK_{ARCH} (red) and HF diet + HEK_{ARCH} (green). The green arrow indicates the therapeutic cell implantation procedure on day 5. Data are shown as mean ± SEM; n = 4 mice per group. Statistical analyses represent unpaired, two-tailed Welch's t-test with a 95% confidence interval, where *$p < 0.05$, ***$p < 0.001$. Source data for this figure is available in the Source Data file.
