# Supplementary material for: A synthetic angiotensin II/ACE2-based hormone shunt controlling experimental hypertension
Source: Nat Commun. 2026 Apr 11;17:5116. doi: 10.1038/s41467-026-71796-z (PMC13247273; doi:10.1038/s41467-026-71796-z)
Supplement: Supplementary file 3 — Description of Additional Supplementary Files [file 41467_2026_71796_MOESM3_ESM.pdf]

<u>Description of Additional Supplementary Files</u>

Supplementary Data 1: Plasmid table.

Supplementary Data 2: Annotated plasmid sequences (.gb).
