# Supplementary material for: A synthetic angiotensin II/ACE2-based hormone shunt controlling experimental hypertension
Source: Nat Commun. 2026 Apr 11;17:5116. doi: 10.1038/s41467-026-71796-z (PMC13247273; doi:10.1038/s41467-026-71796-z)
Supplement: Supplementary file 4 — Supplementary Data 1 [file 41467_2026_71796_MOESM4_ESM.pdf]

Supplementary Data 1 | Plasmids designed and used in this study.

Plasmid	Description	Reference
pAB1303	$P_{hCMVmin}$-driven SS-nLuc expression vector. (MCS-$P_{hCMVmin}$-SS-nLuc-pA)	Bertschi et al., unpubl.
pANT7_cGST	Gateway acceptor plasmid.	ThermoFisher Scientific
pANT7_cGST:hSREBF1	pANT7_cGST containing hSREBF1. (dnasu.org no. HsCD00641470)	Arizona State University, unpubl.
pcDNA3.1(+)	Mammalian expression vector. (P_{hCMV}-MCS-pA)	ThermoFisher Scientific
pcDNA3-sACE2(WT)-Fc(IgA1)	P_{hCMV}-driven stACE2 (IgA1) expression vector. (P_{hCMV}-sACE2-IgA1-pA) (Addgene no. 145154)	[1]
pcDNA3-sACE2(WT)-Fc(IgG1)	P_{hCMV}-driven stACE2 (IgG1) expression vector. (P_{hCMV}-sACE2-IgG1-pA) (Addgene no. 145163)	[1]
pcDNA3-sACE2-WT(732)	P_{hCMV}-driven sACE2 expression vector. (P_{hCMV}-sACE2-pA) (Addgene no. 154098)	[1]
pCK53	P_{CRE}-driven SEAP expression vector. (P_{CRE}-SEAP-pA)	[2]
pCXN2-HA-ATR1-YFP	P_{CAG}-driven hAT_1R expression vector. (P_{CAG}-hAT_1R-HA-YFP-pA) (Addgene no. 101659)	[3]
pFOX12	P_{hCMV}-driven eGFP expression vector. (P_{hCMV}-eGFP-pA)	Fuchs et al., unpubl.
pFOX26	P_{hCMV}-driven citrine-P2A-SEAP expression vector. (P_{hCMV}-citrine-P2A-SEAP-pA)	[4]
pKR32	$P_{NF\kappa B}$-driven SEAP expression vector. ($P_{NF\kappa B}$-SEAP-pA)	[5]
pMX57	P_{NFAT3}-driven SEAP expression vector. (P_{NFAT3}-SEAP-pA)	[6]
pSB100X	Constitutive mammalian SB100X expression vector. (P_{hCMV}-SB100X-pA) (Addgene no. 34879)	[7]
pTS1017	Mammalian $P_{hCMV^{*}-1}$-driven SEAP expression vector. ($P_{hCMV^{*}-1}$-SEAP-pA)	Strittmatter et al., unpubl.
pTS2336	Constitutive mammalian SS-nLuc-Fc expression vector. (P_{hCMV}-SS-nLuc-Fc-pA)	Strittmatter et al., unpubl.
pTS2338	SB100X-specific transposon encoding P_{RPBSA}-driven Ypet and PuroR expression. (ITR- MCS-pA:P_{RPBSA}-Ypet-P2A-PuroR-pA-ITR)	Strittmatter et al., unpubl.

pTS2344	SB100X-specific transposon encoding P_{RPBSA}-driven mRuby and BlastR expression. (ITR-MCS-pA:P_{RPBSA}-mRuby-P2A-BlastR-pA-ITR)	Strittmatter et al., unpubl.
pYL1	Mammalian P_{CSN}-driven SEAP expression vector. (P_{CSN}-SEAP-pA) (GenBank accession no. MH594278)	[8]
pGU23	Mammalian reporter plasmid encoding P_{CSN}-driven SS-nLuc-Fc expression. P_{CSN} was excised from pYL1 with *Mlu*I/*Eco*RI and ligated into pTS2336 restricted with *Mlu*I/*Eco*RI. (P_{CSN}-SS-nLuc-Fc-pA)	This work
pGU52	SB100X-specific transposon encoding P_{CAG}-driven hAT$_1$R expression as well as constitutive P_{RPBSA}-driven mRuby and BlastR expression. P_{CAG}-hAT$_1$R-pA was excised from pCXN2 with *Mlu*I/*Hin*dIII and ligated into pTS2344 restricted with *Mlu*I/*Hin*dIII. (ITR-P_{CAG}-hAT$_1$R-pA:P_{RPBSA}-mRuby-P2A-BlastR-pA-ITR)	This work
pGU133	Mammalian expression vector encoding P_{CSN}-driven stACE2 (IgG1) expression. stACE2 (IgG1) was PCR-amplified from pcDNA3-sACE2(WT)-Fc(IgG1) with oligonucleotides OGU80 (5'CTCGAATTCACCATGACTAGTGGAAGCGGAGCTACTAACTTCAGCCT'3) and OGU81 (5'CTGCAGGATGTATTCACCTGCAGGGTGTCTAGAAAGTCAACCGGTTTTACCCGGAGACAG'3), restricted with *Nhe*I/*Sbf*I and ligated into pGU23 restricted with *Spe*I/*Sbf*I. (P_{CSN}-stACE2 (IgG1)-pA)	This work
pGU137	Mammalian expression vector encoding P_{CSN}-driven stACE2 (IgG1)-P2A-SEAP expression. P2A-SEAP was PCR-amplified from pFOX26 with oligonucleotides OGU82 (5'CACCTTCACCAGCGACACCGGTCACATGGGC'3) and OGU83 (5'AGAGGTTCGGCAGCCTGCAGGGGTCTGCTCGAATCTGCC'3), restricted with *Age*I/*Sbf*I, and ligated into pGU133 restricted with *Age*I/*Sbf*I. (P_{CSN}-stACE2 (IgG1)-P2A-SEAP-pA)	This work
pGU139	SB100X-specific transposon encoding P_{CSN}-driven stACE2 (IgG1)-P2A-SEAP expression as well as constitutive P_{RPBSA}-driven Ypet and PuroR expression. P_{CSN}-stACE2(IgG1)-P2A-SEAP-pA was excised from pGU137 with *Mlu*I/*Sbf*I, and ligated into pTS2338 restricted with *Mlu*I/*Sbf*I. (ITR-P_{CSN}-stACE2 (IgG1)-P2A-SEAP-pA:P_{RPBSA}-Ypet-P2A-PuroR-pA-ITR)	This work
pGU149	Constitutive P_{hCMV}-driven hSREBF1 expression vector. hSREBF1 was restricted with *Spe*I/*Age*I from pANT7_cGST:hSREBF1 (dnasu.org no. HsCD00641470) and ligated into pTS2336 restricted with *Spe*I/*Age*I. (P_{hCMV}-hSREBF1-pA)	This work
pGU227	Mammalian reporter plasmid encoding P_{SRE2}-driven SS-nLuc. SRE$_2$ was assembled by annealing oligonucleotides OGU118 (5'GATCTCACTGCTTACTGCTCGAGAAAATCACCCCACTGCAAACTCCTCCCCCTGA'3) and OGU119 (5' TCGATCAGGGGGAGGAGTTTGCAGTGGGGTGATTTTCTCGAGCAGTAAGCAGTGA'3) and ligated into pAB1303 restricted with *Bgl*II/*Xho*I. (P_{SRE2}-SS-nLuc-pA)	This work

pGU228	Mammalian reporter plasmid encoding P_{SRE4}-driven SS-nLuc. SRE_2 was assembled by annealing oligonucleotides OGU118 (5'GATCTCACTGCTTACTGCTCGAGAAAATCACCCCACTGCAAACTCCTCCCCCTGA'3) and OGU119 (5' TCGATCAGGGGGAGGAGTTTGCAGTGGGGTGATTTTCTCGAGCAGTAAGCAGTGA'3) and ligated into pGU227 restricted with *Bgl*II/*Xho*I. (P_{SRE4}-SS-nLuc-pA)	This work
pGU229	Mammalian reporter plasmid encoding P_{SRE6}-driven SS-nLuc. SRE_2 was assembled by annealing oligonucleotides OGU118 (5'GATCTCACTGCTTACTGCTCGAGAAAATCACCCCACTGCAAACTCCTCCCCCTGA'3) and OGU119 (5' TCGATCAGGGGGAGGAGTTTGCAGTGGGGTGATTTTCTCGAGCAGTAAGCAGTGA'3) and ligated into pGU228 restricted with *Bgl*II/*Xho*I. (P_{SRE6}-SS-nLuc-pA)	This work
pGU232	Mammalian reporter plasmid encoding P_{SRE6}-driven SEAP. SEAP was restricted with *Spe*I/*Hin*dIII from pTS1017 and ligated into pGU229 restricted with *Spe*I/*Hin*dIII. (P_{SRE6}-SEAP-pA)	This work
pGU234	Mammalian expression vector encoding P_{CSN}-driven hSREBF1 expression. hSREBF1 was PCR-amplified from pGU149 with oligonucleotides OGU120 (5'GTTCGAAGCGGAATTCACC'3) and OGU121 (5'CTCAATTCATGAAGCTTCTACAGAGACGGCCGCT'3), restricted with SpeI/*Hin*dIII and ligated into pGU203 restricted with *Spe*I/*Hin*dIII (P_{CSN}-hSREBF1-pA).	This work

Abbreviations: BlastR, blasticidin resistance gene; **cAMP,** cyclic adenosine monophosphate; **citrine**, improved variant of the yellow fluorescent protein; **CRE,** cAMP response element; **eGFP,** enhanced green fluorescent protein; **Fc,** fragment crystallizable region; **HA**, hemagglutinin tag; **hAT$_1$R**, human type-1 angiotensin receptor; **hSREBF1,** human sterol regulatory element binding transcription factor 1; **IgA1,** immunoglobulin A1; **IgG1,** immunoglobulin G1; **ITR,** inverted terminal repeats; **MCS,** multiple cloning site; **mRuby,** red fluorescent protein; **NFAT,** nuclear factor of activated T cells; **NFκB,** nuclear factor kappa-light-chain-enhancer of activated B-cells; **nLuc**, nanoluciferase; **$O_{NF\kappa B}$**, NFκB specific operator; **OTet,** TetR binding site; **pA,** polyadenylation signal, **P2A**, self-cleaving peptide; **P_{CAG}**, Synthetic constitutive promoter combining the early enhancer of P_{hCMV} and the chicken β-actin promoter; **P_{CSN}**, synthetic promoter containing CRE, SRE, and NFAT enhancer elements upstream of $P_{SV40min}$; **PCR**, polymerase chain reaction; **$P_{CRE(n)}$**; $(CRE)_n$-$P_{hCMVmin}$; **P_{hCMV}**, human cytomegalovirus immediate early promoter; **$P_{hCMVmin}$**, minimal P_{hCMV}; **$P_{hCMV*-1}$**, $(OTet)_7$-$P_{hCMVmin}$; **P_{NFAT3}**, $(NFAT)_3$-$P_{hCMVmin}$; **$P_{NF\kappa B}$**, NFκB responsive promoter ($O_{NF\kappa B}$-TA); **P_{RPBSA},** synthetic constitutive mammalian promoter; **$P_{SRE(n)}$,** $(SRE)_n$-$P_{hCMVmin}$; **P_{SV40}**, simian virus 40 promoter; **$P_{SV40min}$,** minimal P_{SV40}; **PuroR,** puromycin resistance gene; **sACE2,** wild-type soluble angiotensin converting enzyme type 2; **SB100X,** hyperactive Sleeping Beauty transposase; **SEAP**, human placental secreted alkaline phosphatase; **SRE,** sterol response element; **SS**, secretion signal; **stACE2,** soluble therapeutic angiotensin converting enzyme 2; **TA,** TATA box; **TetR,** tetracycline repressor; **YFP**,**Ypet,** yellow fluorescent proteins.

References

1. Chan, K.K. et al. Engineering human ACE2 to optimize binding to the spike protein of SARS coronavirus 2. *Science* **369**, 1261-1265 (2020).
2. Kemmer, C. et al. A designer network coordinating bovine artificial insemination by ovulation-triggered release of implanted sperms. *J Control Release* **150**, 23-29 (2011).
3. Inuzuka, T. et al. Attenuation of ligand-induced activation of angiotensin II type 1 receptor signaling by the type 2 receptor via protein kinase C. *Sci Rep* **6**, 21613 (2016).
4. Haellman, V., Strittmatter, T., Bertschi, A., Stücheli, P. & Fussenegger, M. A versatile plasmid architecture for mammalian synthetic biology (VAMSyB). *Metab Eng* **66**, 41-50 (2021).
5. Schukur, L., Geering, B., Charpin-El Hamri, G. & Fussenegger, M. Implantable synthetic cytokine converter cells with AND-gate logic treat experimental psoriasis. *Sci Transl Med* **7**, 318ra201 (2015).
6. Xie, M. et al. β-cell-mimetic designer cells provide closed-loop glycemic control. *Science* **354**, 1296-1301 (2016).
7. Mátés, L. et al. Molecular evolution of a novel hyperactive Sleeping Beauty transposase enables robust stable gene transfer in vertebrates. *Nat Genet* **41**, 753-761 (2009).
8. Liu, Y., Charpin-El Hamri, G., Ye, H. & Fussenegger, M. A synthetic free fatty acid-regulated transgene switch in mammalian cells and mice. *Nucleic Acids Res* **46**, 9864-9874 (2018).
