# Supplementary material for: A synthetic angiotensin II/ACE2-based hormone shunt controlling experimental hypertension
Source: Nat Commun. 2026 Apr 11;17:5116. doi: 10.1038/s41467-026-71796-z (PMC13247273; doi:10.1038/s41467-026-71796-z)
Supplement: Supplementary file 6 — Reporting Summary [file 41467_2026_71796_MOESM6_ESM.pdf]

nature portfolio

Corresponding author(s): Martin Fussenegger

Last updated by author(s): Mar 29, 2026

Reporting Summary

Nature Portfolio wishes to improve the reproducibility of the work that we publish. This form provides structure for consistency and transparency in reporting. For further information on Nature Portfolio policies, see our Editorial Policies and the Editorial Policy Checklist.

Statistics

For all statistical analyses, confirm that the following items are present in the figure legend, table legend, main text, or Methods section.

n/a	Confirmed	
☐	☒	The exact sample size (*n*) for each experimental group/condition, given as a discrete number and unit of measurement
☐	☒	A statement on whether measurements were taken from distinct samples or whether the same sample was measured repeatedly
☐	☒	The statistical test(s) used AND whether they are one- or two-sided<br>*Only common tests should be described solely by name; describe more complex techniques in the Methods section.*
☒	☐	A description of all covariates tested
☐	☒	A description of any assumptions or corrections, such as tests of normality and adjustment for multiple comparisons
☐	☒	A full description of the statistical parameters including central tendency (e.g. means) or other basic estimates (e.g. regression coefficient) AND variation (e.g. standard deviation) or associated estimates of uncertainty (e.g. confidence intervals)
☐	☒	For null hypothesis testing, the test statistic (e.g. *F*, *t*, *r*) with confidence intervals, effect sizes, degrees of freedom and *P* value noted<br>*Give P values as exact values whenever suitable.*
☒	☐	For Bayesian analysis, information on the choice of priors and Markov chain Monte Carlo settings
☒	☐	For hierarchical and complex designs, identification of the appropriate level for tests and full reporting of outcomes
☒	☐	Estimates of effect sizes (e.g. Cohen's *d*, Pearson's *r*), indicating how they were calculated

Our web collection on statistics for biologists contains articles on many of the points above.

Software and code

Policy information about availability of computer code

Data collection	Nikon WF3 + X-light SD confocal microscope was used for confocal microscopy.<br>Tecan M1000 plate reader (TECAN AG, Switzerland) was used for luminescence, fluorescence, and absorbance measurements.<br>BP-2000 tail-cuff machine (Bioseb, France) was used for non-invasive blood pressure measurements.
Data analysis	The data were processed by GraphPad Prism 8 (v 9.2.0, GraphPad Software Inc.) and Microsoft Excel (v 16.51, Microsoft Inc.).

For manuscripts utilizing custom algorithms or software that are central to the research but not yet described in published literature, software must be made available to editors and reviewers. We strongly encourage code deposition in a community repository (e.g. GitHub). See the Nature Portfolio guidelines for submitting code & software for further information.

Data

Policy information about availability of data

All manuscripts must include a data availability statement. This statement should provide the following information, where applicable:

- Accession codes, unique identifiers, or web links for publicly available datasets
- A description of any restrictions on data availability
- For clinical datasets or third party data, please ensure that the statement adheres to our policy

The authors declare that all data supporting the findings of this study are available herein. Source data are provided with this paper. The data generated in this

study are provided in the Supplementary Information/Source Data file. Annotated plasmid sequences are provided in Supplementary Data 2. All original plasmids used in this study are available from the authors.

Research involving human participants, their data, or biological material

Policy information about studies with human participants or human data. See also policy information about sex, gender (identity/presentation), and sexual orientation and race, ethnicity and racism.

Reporting on sex and gender	Sex and gender were not considered in study design.
Reporting on race, ethnicity, or other socially relevant groupings	Race, ethnicity, or other socially relevant groupings were not considered in study design.
Population characteristics	Population characteristics were not considered in study design.
Recruitment	Hypertensive patients donated samples voluntarily.
Ethics oversight	All patients provided written informed consent and the study has been approved by the IRB (IRB00003565, Ethics Commission Northwest- and Central Switzerland).

Note that full information on the approval of the study protocol must also be provided in the manuscript.

Field-specific reporting

Please select the one below that is the best fit for your research. If you are not sure, read the appropriate sections before making your selection.

☒ Life sciences ☐ Behavioural & social sciences ☐ Ecological, evolutionary & environmental sciences

For a reference copy of the document with all sections, see nature.com/documents/nr-reporting-summary-flat.pdf

Life sciences study design

All studies must disclose on these points even when the disclosure is negative.

Sample size	A minimum of 3 replicates were used for each condition for in vitro experiments. For in vivo experiments, a minimum of 4 were used. No statistical methods were used to predetermine sample size for each experiment.
Data exclusions	No data were excluded from the analyses.
Replication	All in vitro data were successfully reproduced at least twice with proper controls to ensure that no external factors (cell viability, transfection efficiency, etc.) affect data accuracy.
Randomization	For in vivo experiments, mice allocation into different groups were randomized.
Blinding	Investigators were blinded to group allocation during data collection and analysis.

Reporting for specific materials, systems and methods

We require information from authors about some types of materials, experimental systems and methods used in many studies. Here, indicate whether each material, system or method listed is relevant to your study. If you are not sure if a list item applies to your research, read the appropriate section before selecting a response.

Materials & experimental systems

n/a	Involved in the study	
☐	☒	Antibodies
☐	☒	Eukaryotic cell lines
☒	☐	Palaeontology and archaeology
☐	☒	Animals and other organisms
☒	☐	Clinical data
☒	☐	Dual use research of concern
☒	☐	Plants

Methods

n/a	Involved in the study	
☒	☐	ChIP-seq
☒	☐	Flow cytometry
☒	☐	MRI-based neuroimaging

Antibodies

Antibodies used	Anti-mouse CD16/32 (93, 101302), anti-mouse CD4 (RM4-5, 100527), anti-mouse CD8β (YTS256.7.7, 126617 and 126633), anti-mouse F4/80 (BM8, 123107), anti-mouse CD25 (PC61, 102011), anti-mouse/human CD44 (IM7, 103006), anti-mouse CX3CR1 (SA011F11, 149009), anti-mouse PD-1 (29F.1A12, 135205), anti-mouse CD11c (N418, 117308), and anti-mouse CD80 (16-10A1, 104734) antibodies were purchased from BioLegend.
Validation	Validated by manufacturer as indicated on the manufacturers' websites.

Eukaryotic cell lines

Policy information about cell lines and Sex and Gender in Research

Cell line source(s)	CELL LINES: Human embryonic kidney (HEK-293T, ATCC: CRL-3216), COS-7 (ATCC: CRL-1651), MDCK (ATCC: CCL-34), BHK-21 (ATCC: CCL-10), NIH/3T3 (ATCC: CRL-1658), HepG2 (ATCC: HB-8065), hMSC-TERT (Simonsen et al., Nature Biotechnology, 2002).
Authentication	All the cell lines used in this study were re-authenticated by the supplier and the authorities of the Department of Biosystems Science and Engineering (D-BSSE) of the ETH Zurich in Basel, Switzerland. The quality of the cells was double-checked by the morphology before start of experiment.
Mycoplasma contamination	The cell lines in this study were tested for mycoplasma and returned negative.
Commonly misidentified lines (See ICLAC register)	N/A.

Animals and other research organisms

Policy information about studies involving animals; ARRIVE guidelines recommended for reporting animal research, and Sex and Gender in Research

Laboratory animals	Male C57BL/6J mice, aged 12 weeks, weighing 24-26 g were purchased from Janvier Labs (53940 Le Genest-Saint-Isle, France)<br><br>Male B6.V-Lepob/JRj (ob/ob) mice (4 weeks old) were obtained from Janvier Labs (France).
Wild animals	N/A.
Reporting on sex	Sex was not considered in study design.
Field-collected samples	N/A.
Ethics oversight	All experiments involving animals were performed according to the directive of the European Community Council (2010/63/EU), approved by the French Republic (project No. DR2018-40v5 and APAFIS #16753) and according to the Swiss animal welfare legislation (art. 18, 141, 30), were approved by the Veterinary Office of Canton Basel-Stadt (GenoMet; national No.: 35863, cantonal No.: 3200).

Note that full information on the approval of the study protocol must also be provided in the manuscript.

Plants

Seed stocks	No plants were involved in this study.
Novel plant genotypes	No plants were involved in this study.
Authentication	No plants were involved in this study.
