# Supplementary material for: A synthetic angiotensin II/ACE2-based hormone shunt controlling experimental hypertension
Source: Nat Commun. 2026 Apr 11;17:5116. doi: 10.1038/s41467-026-71796-z (PMC13247273; doi:10.1038/s41467-026-71796-z)
Supplement: Supplementary file 2 — Peer Review File [file 41467_2026_71796_MOESM2_ESM.pdf]

nature portfolio

Peer Review File

A synthetic angiotensin II/ACE2-based hormone shunt controlling experimental hypertension

Corresponding Author: Professor Martin Fussenegger

This file contains all reviewer reports in order by version, followed by all author rebuttals in order by version.

Version 0:

Reviewer comments:

Reviewer #1

(Remarks to the Author)
Unal et al. report a fully human closed-loop gene circuit (ARCH) for autonomous blood pressure regulation. The authors rewire AT1R into a biosensor that detects angiotensin II (AT-II) and couple it to the expression of a secretion-engineered soluble ACE2 (stACE2). They demonstrate restoration and maintenance of normal blood pressure following implantation of microencapsulated ARCH-transgenic human cells, providing convincing proof-of-concept evidence. Overall, this study represents a significant advance in the application of synthetic biology to chronic disease therapy and offers a promising approach that warrants further investigation. Before publication, I have some minor concerns as follows:

1. Lines 244-249, the AT-II sensor was functional across multiple mammalian cell types, with the strongest response observed in HEK-293T cells. Could the authors speculate on which factors might underlie these differences, such as receptor expression levels or cell-type-specific signaling efficiency?

2. Lines 314-321, the stable monoclonal HEKARCH cell line showed ~300-fold higher AT-II sensitivity compared to transiently transfected cells. Could the authors explain the potential factors underlying this enhanced sensitivity? For example, is this improvement due to increased copy number, higher expression stability, or other cell-intrinsic mechanisms? Clarifying these factors could provide valuable guidance for improving the sensitivity of similar systems in future studies.

3. Lines 294-304: sACE2 is described as a licensed ad hoc drug for the treatment of Covid-19. Given that hypertension is a chronic condition requiring long-term treatment, could the authors discuss the potential risks associated with prolonged ACE2 protein secretion? Although ACE2 expression and secretion are controlled by the AT-II sensor, it would be important for the authors to comment on what levels or duration of AT-II sensor-responsive ACE2 expression could be considered safe.

4. Could the authors provide information on the response kinetics of the system? Specifically, how rapidly can the AT-II sensor–controlled ACE2 expression respond to changes in AT-II levels, and how quickly does this translate into measurable blood pressure reduction?

5. If possible, could the authors verify whether the system is capable of responding to blood samples from hypertensive patients? Demonstrating responsiveness in clinically relevant samples would further support the translational potential and therapeutic relevance of the system.

6. This study demonstrates synthetic gene circuits with potential for treating chronic diseases. To contextualize these findings and highlight their potential applications, the authors may refer to representative previous works, including synthetic gene circuits for treating chronic diseases via inhalation (PMID: 38321056) and for diabetes therapy in large animals (PMID: 31645456, 34608325).

7. The black bars obscure the distribution of individual data points; please consider using a different color to improve visibility. Regarding Fig.1a, the current schematic is overly simplified. It would be helpful to provide a more detailed illustration of the signaling pathway and the operational process of the system.

Reviewer #2

(Remarks to the Author)
In this study, Unal et al. designed a cell-based therapy for hypertension called ARCH (autonomous regulator of chronic hypertension), which utilized human cells co-transfected with type-1 angiotensin receptor (AT1R) and a chimeric promoter (PCSN, CRE-SRE-NFAT-Pmin) driven soluble therapeutic ACE2 (stACE2). Increased blood Angiotensin-II (Ang-II) levels under hypertensive conditions would activate AT1R, which then induce the expression of stACE2 through the chimeric promoter. The authors first tested this system in cultured cells and then tested its therapeutic potential in the Ang-II infusion model of hypertension in mice. The same research group has designed similar closed-loop systems for the treatment of experimental arthritis, obesity, and diabetes, but its application in hypertension is new. However, there is a major concern of utilizing Ang-II levels as the activation switch, as blood Ang-II levels in patients with hypertension vary. Although Ang-II levels are usually elevated in patients with severe essential hypertension, renal hypertension, and malignant hypertension, its levels are usually normal in benign and uncomplicated essential hypertension. Moreover, blood Ang-II levels can be altered by drugs including many anti-hypertensive medications. Thus, the utility of such a system in hypertensive patients is questionable.

In addition, the following concerns are noted about the proposed cell-based therapy:

1. Plasma Ang-1-7 levels should be measured in the in vivo validation experiment.
2. The Ang-II infusion model is an acute, not chronic, model of experimental hypertension.
3. The anti-hypertensive drugs used in Figure 4a affect Ang-II levels in vivo, thus this cell-based (HEK 293) assay cannot rule out the interactions of ARCH with canonical hypertension medications.
4. The tai cuff method is less reliable than radiotelemetry, especially for diastolic blood pressure measurement.
5. What behavioral monitoring was conducted in these mice (line 347)?
6. The authors stated that “the cell-containing capsules were harvested at two weeks post-implantation together with the surrounding peritoneal lavage (line 352-253),” but this is not consistent with how the cells were implanted, line 214-215 “Therapeutic cell capsules were injected subcutaneously (1 mL, ~5 million cells per mouse) on day 4 in the caudal region.”
7. Leukocyte activation markers should be included in the leukocyte profiling (Supplementary Figure 5).
8. A two-tailed t-test is more appropriate than the one-tailed t-test used in this study. What statistical methods were used when more than two groups were compared?
9. Please list the number of mice used in each group in the in vivo experiment.

Reviewer #3

(Remarks to the Author)
Unal et al showed a proof-of-concept therapy based on a synthetic biology approach to treat hypertension. The technology involved the development of a fully human, closed-loop gene circuit named ARCH (Autonomous Regulator of Chronic Hypertension), that leverages the angiotensin II type 1 receptor (AT1R) and a stabilized, secretion-engineered angiotensin-converting enzyme 2 (stACE2), to autonomously sense and regulate blood pressure. This is a highly innovative approach, and experiments clearly showed possible application.

1. It is not clear why the authors included an NFAT response element in the synthetic promoter, because, as depicted in Figure 1C, PNFAT did not respond to Ang II.
2. In supplementary figure 2, the authors showed a basic (panel a), and a booster (panel b) sensor design. It is not clear whether the experiments performed in the manuscript were done using the basic or the booster design.
3. In figure 2b, angiotensin A, angiotensin-(1-9,) and angiotensin-(1-5) should also be tested.
4. In figure 2f, the drugs were added in the presence of Ang II? Because in the figure appears as - AT-II + Drug
5. Only protein levels of stACE2 were measured. Because the enzymatic activity of ACE2 is important for its biological effect, stACE2 activity should also be measured.
6. How stable is the HEKARCH (Stable, monoclonal) cell line? Because synthetic gene circuits may undergo genetic drift or silencing over time.
7. The in vivo experiments were performed using an Ang II-infused mouse model. Because hypertension is multifactorial and not solely Ang II dependent, a different hypertension model should be tested, for instance, on spontaneously hypertensive rats.
8. In the in vivo experiment, a control verifying that the decrease in blood pressure is due to stACE2should be included, i.e., by using an inhibitor of stACE2.
9. In the animals, are the levels of Ang II decreased and the levels of Ang-(1-7) increased?
10. Produces this approach also a reduction of cardiac remodeling, vascular remodeling?

Reviewer #4

(Remarks to the Author)
The study approach is unique and novel model to use ARCH-transgenic human cells and elevate stACE2 to treat AT-II hypertension. In this approach, it is expected that stACE2 will break down (reduce) hypertensive AT-II and increase anti-hypertensive Ang1-7 peptide hormones. However, to validate these mechanisms, some measurements, as follows, are important.

1. Plasma as well as tissue, particularly kidney levels of AT-II and Ang (1-7) will tell whether the levels of these peptide hormones really changed and contributed to lowering the BP. Please measure the levels of these peptides.

2. ACE2 activity, not just the expression, is critical to correlate with the changes in AT-II and Ang(1-7).

3. Blocking/reducing AT1R is known to increase the renin activity, which has independently profibrotic activity via pro-renin receptors. So please measure renin activity in the plasma and the kidney.

4. AT-II model of hypertension is good in animal models to understand the mechanism. For translational purposes, a salt-sensitive model of hypertension could be more helpful.

Version 1:

Reviewer comments:

Reviewer #1

(Remarks to the Author)
They have addressed all my concerns, so I have no further questions.

Reviewer #2

(Remarks to the Author)
The authors did a good job addressing the previous critiques. However, I have the following remaining concerns:
1. Figure 1a shows four pathways downstream of AT1R, but only one pathway is shown in Figure 3a. This is confusing since the broad-spectrum PCSN with combined CRE, SRE, and NFAT operator modules was selected for the ARCH system.
2. Figure 6: In the obesity model, please include regular diet control. What are the methods for Ang-II and Ang-1-7 peptide measurement? What are the Ang-II levels in pg/mL? Are the values consistent with published literature?
3. The plasma Ang-II levels in Figures 6 and 7 are very different. Please explain.
4. The sample size (4 animals per group) is low for the in vivo experiments.
5. Lines 387-388 “Valsartan and ARCH exhibited comparable efficacy in a kinetic assay.” Should it be Valsartan and stACE2 exhibited comparable efficacy in a kinetic assay?

Reviewer #3

(Remarks to the Author)
Unal et al performed most of the suggested changes. However, some concerns still remained:
1. In Figure 3f, Ang II levels were measured as a readout of stACE2 activity. However, a decrease in Ang II levels could also be produced by aminopeptidase II, dipeptidyl aminopeptidase, neprilysin, etc. All these enzymes are present in HEK293 cells. Therefore, I recommend a direct measure of stACE2 enzymatic activity.
2. On page 8, line 390, it should be clearly stated that the hypertension model is an Ang-II-infused mouse model.
3. Incorporate the fig R1 as supplementary data in the manuscript.
4. The quantification methods for Ang II, Ang-(1-7), and renin are not described in methods.
5. Arrows of Figure 6 (a, c, e, g) are not described in the respective legend. In the same figure, why does the addition of an inhibitor of ACE2 reduce the amount of ACE2? ACE2 inhibitors should not reduce plasma ACE2 levels; instead, they should only inhibit its enzymatic activity, thus preventing the conversion of Ang II to Ang-(1-7).

Reviewer #4

(Remarks to the Author)
The manuscript is adequately revised with additional data as asked in the original review. However, this reviewer could not locate the methods used to quantify angiotensin II and angiotensin (1-7) peptides in mice plasma and kidney samples. Method for renin measurement is mentioned under human samples; if the same method is used in animals' samples, please state so.

Version 2:

Reviewer comments:

Reviewer #2

(Remarks to the Author)
I have no additional comments.

Reviewer #3

(Remarks to the Author)
The manuscript is adequately revised with corrections as requested.

Reviewer #4

(Remarks to the Author)
Authors have clarified my concerns and questions and revised the manuscript adequately.

Open Access This Peer Review File is licensed under a Creative Commons Attribution 4.0 International License, which permits use, sharing, adaptation, distribution and reproduction in any medium or format, as long as you give appropriate credit to the original author(s) and the source, provide a link to the Creative Commons license, and indicate if changes were made.
In cases where reviewers are anonymous, credit should be given to 'Anonymous Referee' and the source.
The images or other third party material in this Peer Review File are included in the article’s Creative Commons license, unless indicated otherwise in a credit line to the material. If material is not included in the article’s Creative Commons license and your intended use is not permitted by statutory regulation or exceeds the permitted use, you will need to obtain permission directly from the copyright holder.
To view a copy of this license, visit https://creativecommons.org/licenses/by/4.0/

Response to the reviewers' comments

Reviewer 1

Unal et al. report a fully human closed-loop gene circuit (ARCH) for autonomous blood pressure regulation. The authors rewire AT1R into a biosensor that detects angiotensin II (AT-II) and couple it to the expression of a secretion-engineered soluble ACE2 (stACE2). They demonstrate restoration and maintenance of normal blood pressure following implantation of microencapsulated ARCH-transgenic human cells, providing convincing proof-of-concept evidence. Overall, this study represents a significant advance in the application of synthetic biology to chronic disease therapy and offers a promising approach that warrants further investigation. Before publication, I have some minor concerns as follows:

We thank the reviewer for their positive overall assessment that ARCH provides convincing proof-of-concept and represents a significant advance toward chronic disease therapy. We have addressed all minor concerns, most notably with an expanded discussion of long-term safety under feedback control, additional kinetic characterization, and a demonstration of responsiveness to sera from hypertensive patients, as detailed below.

1. Lines 244-249, the AT-II sensor was functional across multiple mammalian cell types, with the strongest response observed in HEK-293T cells. Could the authors speculate on which factors might underlie these differences, such as receptor expression levels or cell-type-specific signaling efficiency?

The observed differences in Ang-II sensor performance across cell types likely reflect a combination of (i) differences in effective AT_1R/reporter expression levels (driven by variations in cell-type-dependent transfection efficiency and plasmid expression capacity) and (ii) differences in cell-intrinsic coupling efficiency of AT_1R to the downstream cAMP/PKA/CREB cascade that activates the P_{CSN} promoter. HEK-293T cells are widely used for preclinical circuit validation because they typically support robust plasmid-mediated expression and show wide dynamic ranges in transient reporter assays (Yang, et al., *Nature Communications*, 2021, PMID: 34226556; Nissim, et al., *Cell*, 2017, PMID: 29056342; De Carluccio, et al., *Nature Communications*, 2024, PMID: 38632224). More broadly, variable response strengths across cell lines are commonly attributable to differences in transfection efficiency and endogenous cellular components that modulate signaling performance (Wang, et al., *Nature Biomedical Engineering*, 2018, PMID: 31015627; Liu, et al., *Cell*, 2018, PMID: 29937224; Wu, et al., *Nature Communications*, 2020, PMID: 32709899; Mahameed et al., *Nature Biomedical Engineering*, 2025, PMID: 39953326). We have now incorporated this rationale into the revised Results text to clarify why HEK-293T provided the most robust response.

2. Lines 314-321, the stable monoclonal HEKARCH cell line showed ~300-fold higher AT-II sensitivity compared to transiently transfected cells. Could the authors explain the potential factors underlying this enhanced sensitivity? For example, is this improvement due to increased copy number, higher expression stability, or other cell-intrinsic mechanisms? Clarifying these factors could provide valuable guidance for improving the sensitivity of similar systems in future studies.

We thank the reviewer for raising this important point. The marked gain in Ang-II sensitivity in the stable monoclonal HEK_{ARCH} line likely reflects reduced expression

heterogeneity and improved stoichiometric balance relative to transient transfection. In transiently transfected cultures, and likewise in polyclonal stable populations, effective copy number and expression levels vary substantially across cells, and polyclonal integration further introduces site-dependent expression differences due to random genomic insertion. By contrast, monoclonal stable lines originate from single cells and can be screened top-down for favorable circuit properties (high inducibility, low basal activity, and optimal expression balance), which is a standard strategy in circuit engineering and cell-line development (Huang, et al., *Adv. Sci.*, 2024, PMID: 38526196; Unal, et al., *Cell Metab.*, 2025, PMID: 40972574). The above factors are well known to affect transcriptional control parameters, including apparent ligand sensitivity, leakiness, inducibility, and absolute output, as observed in our and others' mammalian gene-circuit work (Mahameed et al., *Nat Biomed Eng*, 2025, PMID: 39953326; Chen et al., *Nat Chem Biol*, PMID: 34782743). We have now clarified this in the revised Results section (lines 357–361).

3. Lines 294-304: sACE2 is described as a licensed ad hoc drug for the treatment of Covid-19. Given that hypertension is a chronic condition requiring long-term treatment, could the authors discuss the potential risks associated with prolonged ACE2 protein secretion? Although ACE2 expression and secretion are controlled by the AT-II sensor, it would be important for the authors to comment on what levels or duration of AT-II sensor-responsive ACE2 expression could be considered safe.

We agree with the reviewer that long-term safety is a central consideration when selecting a therapeutic output for a gene circuit. First, although ACE2 is predominantly membrane-bound, it is physiologically shed and measurable in the circulation as soluble ACE2 in healthy individuals (Batlle et al., *Cell*, 2022, PMID: 35623327). Second, recombinant soluble ACE2 has been clinically evaluated in humans, including in COVID-19 studies, at doses that are approximately three orders of magnitude higher than the maximal output achieved when ARCH is fully activated; the ARCH-level exposure is only 3-4 times greater than endogenous circulating concentrations (Zoufaly et al., *Lancet Respir Med*, 2020, PMID: 33131609; Kawabe et al., *Front Med*, 2022, PMID: 35155493). Importantly, ARCH was intentionally engineered as a feedback-controlled closed-loop system to minimize unnecessary long-term exposure: when Ang-II levels are in the normotensive range, the circuit remains effectively quiescent and transgene output is at background (Fig. 5e, f; blue bars). We have now made this feedback-based safety rationale more explicit in the Discussion (lines 508-510).

4. Could the authors provide information on the response kinetics of the system? Specifically, how rapidly can the AT-II sensor–controlled ACE2 expression respond to changes in AT-II levels, and how quickly does this translate into measurable blood pressure reduction?

We thank the reviewer for highlighting the importance of response kinetics. ARCH is a transcriptionally regulated circuit and therefore operates on transcription, translation, and secretion timescales. Following Ang-II stimulation, secreted transgene output becomes detectable in the culture medium within 4 hours (Fig. 4b). This comment prompted us to add a dedicated kinetic analysis of Ang-II depletion mediated by secreted stACE2, which directly quantifies the time course of Ang-II modulation (new Fig. 3f). Nevertheless, it is important to note that ARCH is intended for angiotensin-dependent chronic hypertension, where dysregulation persists for prolonged periods and requires long-term management rather than minute-scale control (Li et al., *Mol Ther Nucleic Acids*, 2023, PMID: 37449045). We have clarified this intended use case and the relevance of the observed kinetics in the revised text.

5. If possible, could the authors verify whether the system is capable of responding to blood samples from hypertensive patients? Demonstrating responsiveness in clinically relevant samples would further support the translational potential and therapeutic relevance of the system.

We thank the reviewer for this excellent suggestion. We obtained serum samples from hypertensive patients at University Hospital Basel, selecting individuals with high RAS activity, defined as the top 20% for renin and angiotensinogen, and not receiving ACE inhibitors. Using these samples, we now show that HEK_{ARCH} is activated by patients' sera and induces stACE2 output in samples with elevated Ang-II (**new Fig. 7**).

6. This study demonstrates synthetic gene circuits with potential for treating chronic diseases. To contextualize these findings and highlight their potential applications, the authors may refer to representative previous works, including synthetic gene circuits for treating chronic diseases via inhalation (PMID: 38321056) and for diabetes therapy in large animals (PMID: 31645456, 34608325).

As suggested, we have incorporated the cited studies into the Discussion to better contextualize ARCH within the broader landscape of synthetic gene circuits for long-term management of chronic diseases and to support its translational potential.

7. The black bars obscure the distribution of individual data points; please consider using a different color to improve visibility. Regarding Fig.1a, the current schematic is overly simplified. It would be helpful to provide a more detailed illustration of the signaling pathway and the operational process of the system.

Thank you. We have improved the visibility of the control data points throughout the relevant plots and also revised the circuit schematic to provide a more informative representation of ARCH (**revised Fig. 1a**).

Reviewer 2

In this study, Unal et al. designed a cell-based therapy for hypertension called ARCH (autonomous regulator of chronic hypertension), which utilized human cells co-transfected with type-1 angiotensin receptor (AT1R) and a chimeric promoter (PCSN, CRE-SRE-NFAT-Pmin) driven soluble therapeutic ACE2 (stACE2). Increased blood Angiotensin-II (Ang-II) levels under hypertensive conditions would activate AT1R, which then induce the expression of stACE2 through the chimeric promoter. The authors first tested this system in cultured cells and then tested its therapeutic potential in the Ang-II infusion model of hypertension in mice. The same research group has designed similar closed-loop systems for the treatment of experimental arthritis, obesity, and diabetes, but its application in hypertension is new. However, there is a major concern of utilizing Ang-II levels as the activation switch, as blood Ang-II levels in patients with hypertension vary. Although Ang-II levels are usually elevated in patients with severe essential hypertension, renal hypertension, and malignant hypertension, its levels are usually normal in benign and uncomplicated essential hypertension. Moreover, blood Ang-II levels can be altered by drugs including many anti-hypertensive medications. Thus, the utility of such a system in hypertensive patients is questionable.

We thank the reviewer for their thoughtful clinical perspective and for highlighting the mechanistic heterogeneity of hypertension. We agree that Ang-II is not a universal biomarker across all patient subtypes. Accordingly, we have clarified in the manuscript that ARCH is positioned for angiotensin-dependent, RAS-driven hypertension in which Ang-II is persistently elevated. We further emphasize that the well-established inter-individual variability in Ang-II dynamics motivated a closed-loop design for ARCH, which enables autonomous adjustment of stACE2 output in response to patient-specific biomarker levels and therapeutic demand. This closed-loop design also means that ARCH would be compatible with most combination regimens.

In addition, the following concerns are noted about the proposed cell-based therapy:

1. Plasma Ang-1-7 levels should be measured in the in vivo validation experiment.

We agree that a more comprehensive analysis of the angiotensin axis is important to substantiate autonomous, feedback-controlled regulation in vivo. In response, we introduced an additional in vivo setting of RAS dysregulation using a high-fat diet obesity model, in which adipose tissue RAS activation increases Ang-II production and release (Yasue et al., *Am J Hypertens*, 2010, PMID: 20057360). We then quantified key components of the axis in both plasma and kidney, including Ang-II, Ang-(1–7), soluble ACE2, and renin, to directly link ARCH activation and stACE2 output with modulation of the endogenous RAS. These data demonstrate elevation of plasma and renal Ang-II in obese mice and its attenuation following ARCH implantation, accompanied by increased circulating soluble ACE2, a transient plasma Ang-(1–7) increase, and compensatory renin elevation (**new Fig. 6**).

2. The Ang-II infusion model is an acute, not chronic, model of experimental hypertension.

The Ang-II infusion paradigm can be implemented as either an acute or a chronic model, depending on the dosing regimen and exposure time. Acute designs typically use bolus administration or short infusions of the order of minutes to hours, whereas chronic hypertension models commonly employ continuous Ang-II delivery via osmotic minipumps for 1–4 weeks (or longer). Our in vivo experiments used this established chronic infusion format, which has been widely applied to model sustained Ang-II-driven hypertension (Crowley et al., *PNAS*, 2006, PMID: 17090678; Guzik et al., *J Exp Med*, 2007, PMID: 17875676; Dikalova et al., *Circ Res*, 2010, PMID: 20448215).

3. The anti-hypertensive drugs used in Figure 4a affect Ang-II levels in vivo, thus this cell-based (HEK 293) assay cannot rule out the interactions of ARCH with canonical hypertension medications.

We agree with the reviewer that many antihypertensive drugs modulate Ang-II levels in vivo and would therefore influence ARCH indirectly by changing the input signal to the feedback loop. For example, ACE inhibitors reduce Ang-II formation. However, the purpose of Fig. 4a was to address a different question, namely whether commonly used antihypertensives with targets unrelated to ARCH directly interfere with circuit function through molecular cross-reactivity within their therapeutic concentration ranges. In this assay, these agents did not measurably perturb ARCH activity, supporting the compatibility of ARCH with combination regimens. As expected, AT_1R blockers constitute an exception because they prevent Ang-II sensing and therefore shut down the circuit. We have clarified this distinction and rationale in the revised text (lines 385-387).

4. The tail cuff method is less reliable than radiotelemetry, especially for diastolic blood pressure measurement.

We agree that radiotelemetry provides unmatched temporal resolution for blood pressure monitoring. We considered telemetry, but selected tail-cuff plethysmography because it was fit for purpose for the primary question addressed here, namely detecting sustained differences in systolic blood pressure between groups over time. ARCH operates through transcription, translation, and secretion, and its functional response therefore occurs on the order of hours, rather than capturing circadian or beat-to-beat dynamics (**new Fig. 3f**; Chen et al., *Nat Med*, 2015, PMID: 26259032; Li et al., *Nat Commun*, 2022, PMID: 35668093). A particular consideration was that tail-cuff measurement is non-invasive and is recommended for assessing substantial group differences and longitudinal changes in systolic blood pressure, consistent with American Heart Association guidance (Kurtz et al., *Hypertension*, 2005, PMID: 15611363). Given that animals already underwent major procedures in this study, we aimed to minimize additional surgical burden and stress associated with telemetry implantation, which can itself affect cardiovascular readouts (Cantalupo et al., *Nat Med*, 2015, PMID: 26301690; Santisteban et al., *Nat Neurosci*, 2024, PMID: 38049579; Harrison et al., *Hypertension*, 2024, PMID: 37990918). We also implemented standard quality-control measures for tail-cuff reliability, including the use of a BP-2000 system with temperature control and appropriately sized restrainers, and one week of pre-training during acclimatization. These methodological details have now been added to the Methods section (lines 237–240).

5. What behavioral monitoring was conducted in these mice (line 347)?

Animal welfare monitoring was performed in accordance with the Swiss Animal Welfare Act using a protocol-specific, standardized score sheet for each animal, incorporating both physical and behavioral assessments. The monitored parameters included appearance, respiration, eyes, behavior, posture, locomotion, seizure activity, and body weight, each recorded as part of a welfare score to guide intervention if needed. We have now included these monitoring criteria in the Methods section (lines 252–253).

6. The authors stated that “the cell-containing capsules were harvested at two weeks post-implantation together with the surrounding peritoneal lavage (line 352-253),” but this is not consistent with how the cells were implanted, line 214-215 “Therapeutic cell capsules were injected subcutaneously (1 mL, ~5 million cells per mouse) on day 4 in the caudal region.”

We thank the reviewer for their careful reading of the text. The statement regarding intraperitoneal recovery referred to the leukocyte profiling experiment, which was performed as a separate host-compatibility study using intraperitoneal implantation and subsequent peritoneal lavage collection. This was inadvertently conflated with the subcutaneous implantation procedure described for the blood-pressure study in the Methods. We have now corrected and clarified the Methods section accordingly (lines 242–244) to clearly distinguish the two in vivo protocols.

7. Leukocyte activation markers should be included in the leukocyte profiling (Supplementary Figure 5).

We appreciate this suggestion. We repeated the leukocyte profiling experiment and expanded the panel to include commonly used leukocyte activation markers (**new Fig. S6**).

Across the assessed populations, these activation markers were not significantly increased in implanted animals compared with non-implanted controls. Thus, there was no evidence of overt immune activation under the conditions tested.

8. A two-tailed t-test is more appropriate than the one-tailed t-test used in this study. What statistical methods were used when more than two groups were compared?

We thank the reviewer for pointing this out. We have now consistently applied two-tailed statistical testing throughout the study. In vitro comparisons involved two groups and were analyzed using two-tailed t-tests. For the in vivo systolic blood pressure time-course data, we used a linear mixed model in R to account for repeated measurements within animals and to estimate group-wise mean responses over time. Full details are provided in the Methods.

9. Please list the number of mice used in each group in the in vivo experiment.

The group sizes were already specified in the Fig. 5 legend; we have now additionally included the number of animals per group in the Methods to ensure this information is immediately accessible and consistently reported throughout the manuscript.

Reviewer 3

Unal et al showed a proof-of-concept therapy based on a synthetic biology approach to treat hypertension. The technology involved the development of a fully human, closed-loop gene circuit named ARCH (Autonomous Regulator of Chronic Hypertension), that leverages the angiotensin II type 1 receptor (AT1R) and a stabilized, secretion-engineered angiotensin-converting enzyme 2 (stACE2), to autonomously sense and regulate blood pressure. This is a highly innovative approach, and experiments clearly showed possible application.

We thank the reviewer for their positive overall assessment and for recognizing the innovation and potential applicability of ARCH as a fully human, closed-loop gene circuit for autonomous blood-pressure regulation. In response to the comments, we have further strengthened the manuscript with additional in vivo mechanistic validation of RAS modulation, including expanded angiotensin-axis profiling and pharmacological inhibition of ACE2 activity, and we have also provided further support for translational relevance by testing responsiveness to sera from hypertensive patients. We address each point in detail below.

1. It is not clear why the authors included an NFAT response element in the synthetic promoter, because, as depicted in Figure 1C, PNFAT did not respond to Ang II.

We selected the synthetic P_{CSN} promoter because it integrates three repeats of CRE, SRE, and NFAT operator modules and provides robust, low-leakage, highly inducible transcriptional control across multiple signaling pathways. P_{CSN} was previously engineered and characterized as a broadly responsive promoter architecture for mammalian gene circuits (Liu et al., *Nucleic Acids Res*, 2018, PMID: 30219861) and has shown superior performance in diverse cascade settings (Mahameed et al., *Nat Commun*, 2022, PMID: 36446786; Stefanov et al., *Small*, 2022, PMID: 36084222). Consistent with these reports, P_{CSN} also provided strong inducible control downstream of AT1R-mediated cAMP signaling in the ARCH context.

2. In supplementary figure 2, the authors showed a basic (panel a), and a booster (panel b) sensor design. It is not clear whether the experiments performed in the manuscript were done using the basic or the booster design.

All experiments reported in this manuscript were performed using the core ARCH configuration without the booster module, as stated in the Results (lines 336–338). We included the booster plug-in only as an optional design extension to illustrate a modular topology that could be readily integrated to increase gain if required in future applications, but it is not necessary for the conclusions or in vivo performance demonstrated here.

3. In figure 2b, angiotensin A, angiotensin-(1-9,) and angiotensin-(1-5) should also be tested.

We thank the reviewer for this suggestion. We have now expanded the specificity panel to include Ang-A, Ang-(1–9), and Ang-(1–5), thereby providing a more comprehensive assessment of angiotensin analog responsiveness (**revised Fig. 2a, b**).

4. In figure 2f, the drugs were added in the presence of Ang II? Because in the figure appears as - AT-II + Drug

In **Fig. 2f**, the indicated drugs were applied instead of Ang-II (that is, Ang-II was omitted) to test whether they could directly activate the AT_1R/P_{CSN} reporter axis. We have revised the figure legend to state this explicitly.

5. Only protein levels of stACE2 were measured. Because the enzymatic activity of ACE2 is important for its biological effect, stACE2 activity should also be measured.

We agree that more comprehensive angiotensin-axis profiling would strengthen the mechanistic basis for autonomous, feedback-controlled regulation. Accordingly, in a new in vivo experiment we quantified circulating stACE2 together with Ang-II and Ang-(1–7) (**new Fig. 6**). stACE2 levels increased in the treatment group in parallel with attenuation of Ang-II, consistent with enzymatic Ang-II cleavage in vivo. This mechanism aligns well with the in vitro time-resolved Ang-II depletion kinetics we now include (**new Fig. 3f**) and also with established ACE2 biology within the vasoprotective RAS axis (Paz Ocaranza et al., *Nat Rev Cardiol*, 2020, PMID: 31427727).

6. How stable is the HEKARCH (Stable, monoclonal) cell line? Because synthetic gene circuits may undergo genetic drift or silencing over time.

We agree this is an important issue. We have now assessed HEK_{ARCH} circuit performance across multiple passage numbers (P10, P20, and P30) and observed stable inducible function across this range (**new Fig. 3c**), supporting the robustness of the engineered monoclonal line. This passage window is consistent with commonly used industrial qualification ranges for HEK-293T-derived production cell lines, which have frequently been validated over 20–40 passages without evidence of genetic drift or functional decline (Grieger et al., *Mol Ther*, 2015, PMID: 26437810).

7. The in vivo experiments were performed using an Ang II-infused mouse model. Because hypertension is multifactorial and not solely Ang II dependent, a different hypertension model should be tested, for instance, on spontaneously hypertensive rats.

We agree that Ang-II is not a universal biomarker across all hypertension subtypes, and have clarified in the manuscript that ARCH is positioned for angiotensin-dependent, RAS-driven hypertension. As the reviewer kindly suggested, we introduced an additional disease-relevant model of obesity-associated RAS dysregulation, in which adipose RAS activation increases Ang-II production (Yasue et al., *Am J Hypertens*, 2010, PMID: 20057360). In this model, ARCH implantation reduced elevated plasma and renal Ang-II, and the results directly support the expected Ang-II-dependent mechanism (**new Fig. 6**). In addition, we performed a negative-control experiment in an Ang-II-independent hypertension setting using the spontaneously hypertensive BPH/2 strain, which has been reported to exhibit neurogenic, RAS-independent hypertension (Jackson et al., *Front Physiol*, 2019, PMID: 31681017; Jackson et al., *Hypertens Res*, 2020, PMID: 32427944). As expected for a circuit that acts through the Ang-II/AT_1R axis, ARCH had no apparent effect on the blood pressure profile in BPH/2 mice (**Fig. R1** below). Because this negative-control outcome is expected and does not change the interpretation of the mechanistic data provided in **new Fig. 6**, we did not include it in the revised manuscript. We would, however, be happy to incorporate this dataset into the Supplementary Information if the editor or reviewers consider it useful.

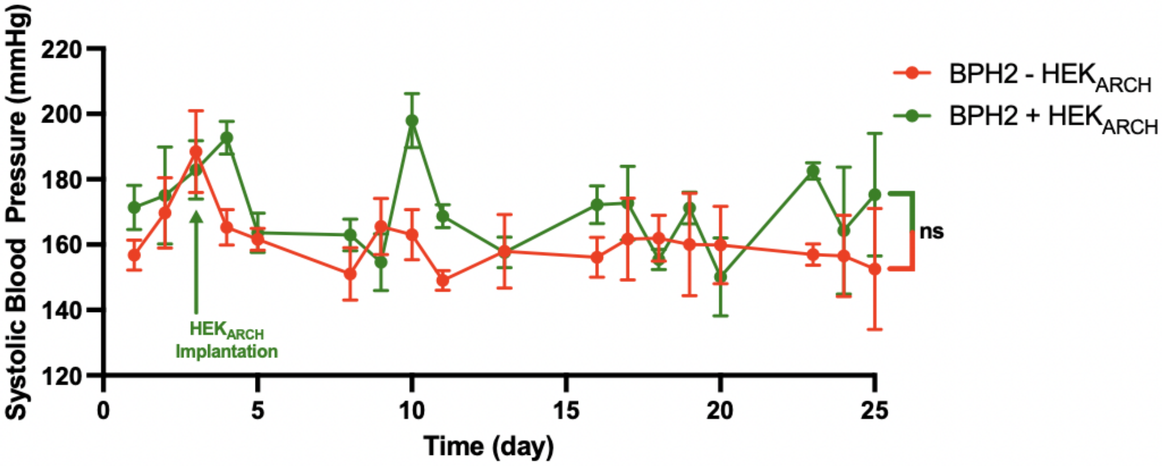

Figure R1 | ARCH in BPH/2 mice. ARCH is designed to regulate Ang-II-dependent hypertension via the Ang-II/AT_1R axis and therefore is not expected to affect neurogenic, RAS-independent hypertension. Consistent with this specificity, ARCH implantation did not significantly change systolic blood pressure in the spontaneously hypertensive BPH/2 strain. Statistical analysis was performed by two-way ANOVA. Data are shown as mean ± SEM; n = 4; ns, not significant.

8. In the in vivo experiment, a control verifying that the decrease in blood pressure is due to stACE2should be included, i.e., by using an inhibitor of stACE2.

We thank the reviewer for this excellent suggestion. We have now added an in vivo pharmacological control using the ACE2 inhibitor MLN-4760. In the new experimental group, mice received ARCH implants and two bolus doses of MLN-4760. Inhibition of ACE2 activity transiently abolished the ARCH-mediated reduction in Ang-II, providing clear evidence that Ang-II attenuation is driven by stACE2 enzymatic activity (**new Fig. 6**).

9. In the animals, are the levels of Ang II decreased and the levels of Ang-(1-7) increased?

We have now performed longitudinal profiling of key RAS components, including circulating stACE2, Ang-II, and Ang-(1–7) (**new Fig. 6**). In the treatment group, increased stACE2 coincided with sustained attenuation of Ang-II and an early increase in Ang-(1–7),

consistent with ACE2-mediated conversion of Ang-II within the vasoprotective RAS axis (Paz Ocaranza et al., *Nat Rev Cardiol*, 2020, PMID: 31427727). The Ang-(1–7) difference was transient, which is consistent with downstream metabolism of Ang-(1–7) into peptides such as Ang-(1–5) and alamandine. As ARCH acts upstream by reducing Ang-II, it is not expected to directly modulate these downstream processing steps (**revised Fig. 2a**).

10. Produces this approach also a reduction of cardiac remodeling, vascular remodeling?

We thank the reviewer for raising this point. Elevated Ang-II has been implicated not only in hypertension but also in additional pathophysiological processes, including cardiovascular remodeling, atherogenesis, renal fibrosis, insulin resistance, and tumor angiogenesis (Domenighetti et al., *Hypertension*, 2005, PMID: 15998712; Lovren et al., *Am J Physiol Heart Circ Physiol*, 2008, PMID: 18660448; Mezzano et al., *Hypertension*, 2001, PMID: 11566946; Underwood, *Curr Hypertens Rep*, 2013, PMID: 23242734; Egami et al., *J Clin Invest*, 2003, PMID: 12840060). Accordingly, sustained Ang-II attenuation through ARCH could, in principle, confer broader vasoprotective effects beyond blood-pressure control, including potential mitigation of Ang-II-driven cardiovascular remodeling. This would be an interesting direction for future work.

Reviewer 4

The study approach is unique and novel model to use ARCH-transgenic human cells and elevate stACE2 to treat AT-II hypertension. In this approach, it is expected that stACE2 will break down (reduce) hypertensive AT-II and increase anti-hypertensive Ang1-7 peptide hormones. However, to validate these mechanisms, some measurements, as follows, are important.

We thank the reviewer for the constructive assessment and helpful suggestions. We have addressed the key points by strengthening in vivo mechanistic validation of RAS modulation, including expanded angiotensin-axis profiling and introduction of an ACE2-inhibitor control, and by clarifying the experimental design and interpretation throughout the manuscript. We respond to each point in detail below.

1. Plasma as well as tissue, particularly kidney levels of AT-II and Ang (1-7) will tell whether the levels of these peptide hormones really changed and contributed to lowering the BP. Please measure the levels of these peptides.

We agree that broader profiling of the angiotensin axis is important to substantiate autonomous, feedback-controlled regulation in vivo. In response, we introduced an additional disease-relevant model of RAS dysregulation using high-fat diet–induced obesity, in which adipose RAS activation increases Ang-II production (Yasue et al., *Am J Hypertens*, 2010, PMID: 20057360). We then quantified key RAS components in both plasma and kidney, including Ang-II, Ang-(1–7), soluble ACE2 (as a readout of stACE2 output), and renin. These measurements directly link ARCH activation to attenuation of elevated Ang-II, accompanied by an early Ang-(1–7) increase and compensatory renin elevation **(new Fig. 6)**.

2. ACE2 activity, not just the expression, is critical to correlate with the changes in AT-II and Ang(1-7).

To address this important point, we added a pharmacological control using the ACE2 inhibitor MLN-4760. In the new experimental group, mice received ARCH implants and two bolus doses of MLN-4760. ACE2 inhibition transiently abrogated the ARCH-mediated reduction in Ang-II, providing evidence that Ang-II attenuation is driven by stACE2 enzymatic activity (**new Fig. 6**).

3. Blocking/reducing AT1R is known to increase the renin activity, which has independently profibrotic activity via pro-renin receptors. So please measure renin activity in the plasma and the kidney.

Indeed, suppression of AT_1R signaling is known to trigger compensatory renin release from juxtaglomerular cells, leading to increased circulating renin levels (Belyea et al., *Clin Sci*, 2023, PMID: 36503993; Nagalakshmi et al., *Acta Physiol*, 2023, PMID: 37309075). Consistent with this physiology, ARCH implantation increased renin concentrations in both plasma and kidney relative to disease controls (**new Fig. 6g, h**). We agree that elevated renin could, in principle, engage (pro)renin receptor–linked, Ang-II-independent signaling pathways, including profibrotic responses. However, Ang-II itself is the major driver of fibrosis via AT_1R-dependent induction of profibrotic mediators, and sustained Ang-II attenuation is a central therapeutic strategy for preventing end-organ damage in RAS-driven disease (Brenner et al., *N Engl J Med*, 2001, PMID: 11565518; Lewis et al., *N Engl J Med*, 2001, PMID: 11565517). We have clarified this compensatory renin response and its interpretation in the revised text and added an explicit statement to the Discussion highlighting this consideration for future long-term evaluation (lines 494–497).

4. AT-II model of hypertension is good in animal models to understand the mechanism. For translational purposes, a salt-sensitive model of hypertension could be more helpful.

We thank the reviewer for this suggestion. We agree that salt-sensitive hypertension is clinically important. However, high salt intake has been reported to lower circulating Ang-II while promoting Ang-II hypersensitivity (Crestani et al., *Vascul Pharmacol*, 2014, PMID: 24321189). Since ARCH is an Ang-II-sensing, Ang-II–degrading closed-loop circuit designed to respond to elevated Ang-II, we consider a salt-sensitive model would not be suitable as a primary model to test ARCH. We have clarified this in the manuscript to avoid potential misunderstanding. Instead, we selected an obesity-associated RAS dysregulation model, in which Ang-II elevation is a prominent feature, to further evaluate ARCH in a disease-relevant translational context (**new Fig. 6**).

Reviewer #1 (Remarks to the Author):

They have addressed all my concerns, so I have no further questions.

Thank you.

Reviewer #2 (Remarks to the Author):

The authors did a good job addressing the previous critiques. However, I have the following remaining concerns:

Thank you. We addressed the remaining concerns below.

1. Figure 1a shows four pathways downstream of AT1R, but only one pathway is shown in Figure 3a. This is confusing since the broad-spectrum PCSN with combined CRE, SRE, and NFAT operator modules was selected for the ARCH system.

To enhance clarity, we retained the detailed AT_1R downstream signaling map in **Fig. 1a** as a conceptual overview. **Fig. 3a** serves a different purpose and was intentionally streamlined to highlight the ARCH circuit architecture and the broad-spectrum P_{CSN} promoter (integrating CRE, SRE, and NFAT operator modules), rather than re-drawing the full upstream signaling network. The **Fig. 3** legend now explicitly states that P_{CSN} integrates these signaling inputs, and the label "Rewired signalling" is used in the schematic.

2. Figure 6: In the obesity model, please include regular diet control. What are the methods for Ang-II and Ang-1-7 peptide measurement? What are the Ang-II levels in pg/mL? Are the values consistent with published literature?

Thank you for your feedback. We performed a regular-diet control experiment in the obesity model and found that normal chow (NC)-fed mice did not show elevated plasma Ang II levels. As these animals did not exhibit the obesity-associated increase in Ang II that the ARCH circuit is designed to counteract, the control dataset was not shown in the previous version. In response to the reviewer's request, we now provide these data as new **Supplementary Fig. 8**.

Ang II was quantified using our cell-based HEK_{ARCH} reporter assay, whereas Ang-(1–7) and renin were quantified using corresponding commercial ELISA kits. These measurement procedures are now described in the Methods section (lines 224–234). In addition, as requested, Ang II is now reported in absolute units in the **revised Figs. 6a-b, 7e**. Across groups, plasma Ang II values were in the range of approximately 20–200 pg/mL, which is within the operating range of our reporter assay and is consistent with published reports using comparable experimental settings and quantification approaches (Yiannikouris, *Hypertension*, 2013, PMID: 23108647; Visniauskas, *AJP-Heart*, 2023, PMID: 36930656). We also note that absolute circulating RAS peptide concentrations can vary substantially across studies, often over orders of magnitude, depending on sample handling, extraction, and assay platform (e.g., immunoassay vs. LC–MS/MS) as well as cohort characteristics (Gurley et al., *JCI*, 2006, PMID: 16878172; Gomolak et al., *Front Physiol*, 2014, PMID: 25400581; Santos et al., *Life Sci*, 2014, PMID: 25219880). Accordingly, we emphasize relative differences within the same experimental and analytical framework as the most directly interpretable readout.

3. The plasma Ang-II levels in Figures 6 and 7 are very different. Please explain.

Relative luminescence units (RLU) are arbitrary units intended to resolve differences within a given experiment and panel. Accordingly, RLU values from different figures should not be compared quantitatively because they depend on background signal, instrument settings, data processing, and independent experimental runs. In **Fig. 7e**, the RLU axis was additionally rescaled to facilitate visualization alongside the clinical RFU readouts. To avoid any ambiguity, and in line with point 2 above, we now provide plasma Ang II levels in absolute units in the **revised Figs. 6a-b, 7e.**

4. The sample size (4 animals per group) is low for the in vivo experiments.

In accordance with European legislation (Directive 2010/63/EC) and the corresponding national regulations governing animal experimentation in the EU, the UK, and Switzerland, animal experiments are conducted under the principles of replacement, reduction, and refinement (3R), with the aim of using the minimum number of animals required to achieve the scientific objective. For each animal license, group sizes are predefined and justified during the approval process based on power calculations that incorporate the expected effect size and variance. Accordingly, group sizes in the range of $3 \leq n \leq 6$ are widely used and accepted for mouse in vivo studies in high-impact research, including in vivo assessments of synthetic biology and protein-based interventions. Representative examples include Vicent et al., *Science*, 2023, PMID: 37824640; Roybal et al., *Cell*, 2016, PMID: 26830879; Reddy et al., *Science*, 2024, PMID: 39636990; Cohen et al., *Science Translational Medicine*, 2026, PMID: 41637528; Franko et al., *Nature Communications*, 2025, PMID: 41198646; Xue et al., *Advanced Science*, 2025, PMID: 40899507; Lin et al., *Nature Nanotechnology*, 2025, PMID: 40325210; Huang et al., *NAR*, 2025, PMID: 40114374; Mahameed et al., *Nature Biomedical Engineering*, 2025, PMID: 39953326. Importantly, the observed effects were consistent across animals within each group, and statistical analyses were performed as described in the Methods. Given the prospective ethical approval with predefined group sizes and the consistency of the observed effects, we believe the current cohorts were sufficient to address the study objectives.

5. Lines 387-388 "Valsartan and ARCH exhibited comparable efficacy in a kinetic assay." Should it be Valsartan and stACE2 exhibited comparable efficacy in a kinetic assay?

The sentence has been reworded for clarity to explicitly refer to the ARCH-mediated stACE2 effector, thereby avoiding any ambiguity between the device (ARCH) and its therapeutic output (stACE2) (lines 403-404).

Reviewer #3 (Remarks to the Author):

Unal et al performed most of the suggested changes. However, some concerns still remained:

Thank you. We addressed the remaining concerns below.

1. In Figure 3f, Ang II levels were measured as a readout of stACE2 activity. However, a decrease in Ang II levels could also be produced by aminopeptidase II, dipeptidyl aminopeptidase, neprilysin, etc. All these enzymes are present in HEK293 cells. Therefore, I recommend a direct measure of stACE2 enzymatic activity.

Thank you for your suggestion. We agree that Ang II depletion alone is not fully specific because additional peptidases present in HEK293 cells can also contribute to Ang II turnover. In the previous version, we therefore relied on the combined evidence of stACE2 production together with Ang II depletion and the consequent increase in Ang-(1–7). In response to your recommendation, we now also provide a direct measurement of enzymatic activity for the recombinantly produced stACE2 fusion protein using a fluorometric ACE2 activity assay (**new Supplementary Fig. 3).**

2. On page 8, line 390, it should be clearly stated that the hypertension model is an Ang-II-infused mouse model.

The text has been revised to explicitly state that the hypertension model is an Ang II-infused mouse model (lines 406-410).

3. Incorporate the fig R1 as supplementary data in the manuscript.

In the previous revision, we provided Fig. R1 in the rebuttal as a negative-control dataset in an Ang II-independent hypertension setting (spontaneously hypertensive BPH/2 strain), which has been reported to exhibit neurogenic, RAS-independent hypertension (Jackson et al., *Front Physiol*, 2019, PMID: 31681017; Jackson et al., *Hypertens Res*, 2020, PMID: 32427944). As expected for a circuit designed to act specifically through the Ang II/AT_1R axis, ARCH had no measurable effect on the blood-pressure profile in this model. This outcome is therefore a predictable null result that follows directly from the defined input dependency of the system and does not add any mechanistic insight. Given the substantial expansion of the dataset across two rounds of revision (**new Figs. 3f, 6a-h**, **7a-f**, and **Supplementary Figs. 3a-b**, **7**, and **8**), we prefer to keep the Supplementary Information focused on experiments that directly support the ARCH mechanism and therapeutic conclusions, and have therefore not incorporated Fig. R1, as we believe it would simply increase manuscript volume without strengthening any conclusion.

4. The quantification methods for Ang II, Ang-(1-7), and renin are not described in methods.

Thank you for pointing this out. Ang II was quantified using our cell-based HEK_{ARCH} reporter assay, whereas Ang-(1–7) and renin were quantified using corresponding commercial ELISA kits. These quantification procedures have now been added to the Methods section (lines 224–234).

5. Arrows of Figure 6 (a, c, e, g) are not described in the respective legend. In the same figure, why does the addition of an inhibitor of ACE2 reduce the amount of ACE2? ACE2 inhibitors should not reduce plasma ACE2 levels; instead, they should only inhibit its enzymatic activity, thus preventing the conversion of Ang II to Ang-(1-7).

The arrows in **Fig. 6a,c,e,g** indicate the timing of key experimental steps. To improve interpretability, we have expanded the description and now annotate this information directly in the respective panels in addition to the figure legend (**revised Fig. 6a,c,e,g**).

Regarding the ACE2 inhibitor, MLN4760 is a competitive ACE2 inhibitor that blocks catalytic activity. In addition, MLN4760 binding has been reported to induce conformational changes in soluble ACE2 that can influence non-catalytic interactions and, depending on the assay format, may affect antibody-based detection of soluble ACE2 (Towler et al., *J Biol Chem*,

2004, PMID: 14754895; Nami et al., *J Hypertens*, 2021, PMID: 34188005; Trozzi et al., *Org Biomol Chem*, 2022, PMID: 35420112). We therefore infer that the reduced signal observed in the presence of MLN4760 reflects altered detectability/assay readout rather than a true reduction in ACE2 abundance. This clarification has been added to the main text (lines 364-369), supported by **new Supplementary Fig. 3**, and the y-axis label has been adjusted accordingly (**revised Fig. 6c**).

Reviewer #4 (Remarks to the Author):

The manuscript is adequately revised with additional data as asked in the original review. However, this reviewer could not locate the methods used to quantify angiotensin II and angiotensin (1-7) peptides in mice plasma and kidney samples. Method for renin measurement is mentioned under human samples; if the same method is used in animals' samples, please state so.

Thank you for pointing this out. Ang II was quantified using our cell-based HEK$_{ARCH}$ reporter assay, whereas Ang-(1–7) and renin were quantified using corresponding commercial ELISA kits for mouse samples. The renin measurement in animals was performed using the same ELISA-based method described for human samples. ACE2 activity was measured using a fluorometric ACE2 activity assay. These quantification procedures have now been explicitly added to the Methods section (lines 224-234).

Reviewer #1 (Remarks to the Author): (Previous round)

They have addressed all my concerns, so I have no further questions.

Thank you.

Thank you.

Thank you.

Thank you.
